# Experimental Neuromyelitis Optica Induces a Type I Interferon Signature in the Spinal Cord

**DOI:** 10.1371/journal.pone.0151244

**Published:** 2016-03-18

**Authors:** Satoru Oji, Eva-Maria Nicolussi, Nathalie Kaufmann, Bleranda Zeka, Kathrin Schanda, Kazuo Fujihara, Zsolt Illes, Charlotte Dahle, Markus Reindl, Hans Lassmann, Monika Bradl

**Affiliations:** 1 Department of Neuroimmunology, Center for Brain Research, Medical University Vienna, Vienna, Austria; 2 Clinical Department of Neurology, Innsbruck Medical University, Innsbruck, Austria; 3 Departments of Multiple Sclerosis Therapeutics and Neurology, Tohoku University Graduate School of Medicine, Sendai, Japan; 4 Department of Neurology, University of Southern Denmark, Odense, Denmark; 5 Department of Clinical Immunology and Transfusion Medicine and Department of Clinical and Experimental Medicine, Linköping University, Linköping, Sweden; University of Düsseldorf, GERMANY

## Abstract

Neuromyelitis optica (NMO) is an acute inflammatory disease of the central nervous system (CNS) which predominantly affects spinal cord and optic nerves. Most patients harbor pathogenic autoantibodies, the so-called NMO-IgGs, which are directed against the water channel aquaporin 4 (AQP4) on astrocytes. When these antibodies gain access to the CNS, they mediate astrocyte destruction by complement-dependent and by antibody-dependent cellular cytotoxicity. In contrast to multiple sclerosis (MS) patients who benefit from therapies involving type I interferons (I-IFN), NMO patients typically do not profit from such treatments. How is I-IFN involved in NMO pathogenesis? To address this question, we made gene expression profiles of spinal cords from Lewis rat models of experimental neuromyelitis optica (ENMO) and experimental autoimmune encephalomyelitis (EAE). We found an upregulation of I-IFN signature genes in EAE spinal cords, and a further upregulation of these genes in ENMO. To learn whether the local I-IFN signature is harmful or beneficial, we induced ENMO by transfer of CNS antigen-specific T cells and NMO-IgG, and treated the animals with I-IFN at the very onset of clinical symptoms, when the blood-brain barrier was open. With this treatment regimen, we could amplify possible effects of the I-IFN induced genes on the transmigration of infiltrating cells through the blood brain barrier, and on lesion formation and expansion, but could avoid effects of I-IFN on the differentiation of pathogenic T and B cells in the lymph nodes. We observed that I-IFN treated ENMO rats had spinal cord lesions with fewer T cells, macrophages/activated microglia and activated neutrophils, and less astrocyte damage than their vehicle treated counterparts, suggesting beneficial effects of I-IFN.

## Introduction

Neuromyelitis optica (NMO) is an acute inflammatory disease of the central nervous system (CNS) which predominantly affects spinal cord and optic nerves, and causes severe, often necrotic lesions characterized by primary astrocyte destruction and secondary myelin loss [1]. In the serum of most, but not all NMO patients, pathogenic autoantibodies against the water channel aquaporin 4 (AQP4) on astrocytes are found [2,3]. While there is currently no cure for this disease, most patients profit from therapies with immunosuppressive corticosteroids, from plasmapheresis removing their pathogenic antibodies from the serum, or from B cell depletion [4]. Surprisingly, NMO patients show peculiar responses to treatment strategies involving type I interferons (I-IFN) like interferon-alpha (IFN-α) or interferon-beta (IFN-β), which sets them clearly apart from MS patients usually benefitting from such therapies [5–9]. Often, NMO patients do not profit from I-IFN therapy [10–12], but there are outliers in response: some patients clearly improve [12,13], while others dramatically deteriorate [6,9,14]. Similarly disparate are observations from experimental studies indicating that type I interferons (I-IFN) did either limit [15], promote [16] or not affect [17] the size of lesions with AQP4 loss. What could be the reason for these findings? To address this question, we studied gene expression patterns in spinal cords of Lewis rats with experimental neuromyelitis optica (ENMO), with experimental autoimmune encephalomyelitis (EAE), or without CNS inflammation, and studied spinal cord lesions in ENMO animals treated at the onset of lesion formation with I-IFN.

## Material and Methods

### Animals

Lewis rats (7–8 weeks old) were obtained from Charles River Wiga (Sulzfeld, Germany). They were housed in the Decentral Facilities of the Institute for Biomedical Research (Medical University Vienna) under standardized conditions. The experiments were approved by the Ethic Commission of the Medical University Vienna and performed with the license of the Austrian Ministery for Science and Research.

### Sources and characterization of patient-derived immunoglobulin preparations

In this study, two different types of immunoglobulin preparations were used.

First, NMO-IgG preparations containing pathogenic AQP4-specific antibodies. These derived from therapeutic plasmapheresates or serum of four different patients (“J0”, “NMO-IgG9”, “Sweden-1” and “pt1”). The NMO-IgGs were essentially prepared and purified as described [18], adjusted to an IgG concentration of 10mg/ml, and gave equal results. The use of the plasmapheresates for research was approved by the Ethics Committee of Tohoku University School of Medicine (No. 2007–327), and by the Regional and National Ethical Committees of Hungary (3893.316-12464/KK4/2010 and 42341-2/2013/EKU) and Sweden (2013/153-31 Linköping).

Secondly, a commercially available normal human IgG preparation (Subcuvia™, Baxter, Vienna), which was used as a negative control in a concentration of 10 mg/ml.

### Gene expression analysis

#### Tissue selection

The spinal cord sections studied were formaldehyde-fixed and paraffin-embedded (FFPE), and derived from Lewis rats of an experimental series described in detail before [18]. These animals had been injected with MBP-specific T cells and NMO-IgG derived from patient J0 [18] (ENMO), with MBP-specific T cells and human control IgG (EAE_coI_), with MBP-specific T cells and PBS (EAE_coP_), with NMO-IgG only, or with human IgG only, and had been sacrificed on day 5 after the injection of T cells (= day 1 after injection of antibodies or PBS). Animals which were left completely untreated were included as healthy controls.

#### RNA isolation and probe preparations

This was essentially done as described [19]. Briefly, 25 spinal cord sections/animal of 5 different animals per experimental group were used. In addition, we used spinal cord cross sections of three healthy controls. The spinal cord sections covered the entire neuraxis. 6–7 μm-thick tissue sections were pooled in RNAse free tubes and deparaffinated with Xylol. Then, total RNA was isolated, the mRNA contained in the isolate was transcribed to cDNA, and one round of RNA amplification and cDNA production was performed, using for all steps the Paradise® Reagent System (Arcturus, USA) according to the instructions of the manufacturer.

#### Microarray analysis

The cDNA was sent to ImaGenes (Berlin, Germany) for microarray analysis using 4x44 K Multiplex whole rat genome microarrays (Agilent G4131F). The raw microarray data were subjected to quantile normalizations prior to comparison between groups and calculation of fold changes in expression. The normalized signal intensities were in the range of 2–163000. The Gene expression data were deposited in the GEO database (GSE73411).

#### Data analysis

In a first round of data analysis, we only considered genes which were upregulated in ENMO compared to any other control group, and which had normalized signal intensities > 100. Then, we calculated (1) the fold changes of ENMO: EAE, in which EAE represented the mean of EAE_coI_ and EAE_coP_, and (2) the fold changes of all T cell mediated diseases (mean value of normalized signal intensities (NSI) of ENMO, EAE_coI_ and EAE_coP_): all non-inflammatory controls (mean value of NSI from NMO-IgG only, IgG only and healthy controls). In further rounds of data analysis, we did no longer use a threshold of NSIs (when we searched for differentially expressed I-IFN response genes), and also considered genes which were downregulated in ENMO compared to any other control group (when we studied astrocyte-related genes).

### Confirmation of microarray data by quantitative real-time polymerase chain reactions (qPCR)

For qPCR reactions, EAE and ENMO was induced essentially as described [18]. Unless otherwise noted, 3 Lewis rats / experimental group were used. The animals were injected with MBP-specific T cells and NMO-IgG (ENMO), with MBP-specific T cells and human control IgG (EAE_coI_), with MBP-specific T cells and PBS (EAE_coP_), with NMO-IgG only (n = 2 rats), or with human IgG only. 3 PBS-treated animals served as healthy controls. All animals were sacrificed by CO_2_ inhalation. The spinal cords were dissected, and RNA was prepared and transcribed to cDNA essentially as described [20], using M-MLV Reverse transcriptase (Promega, Mannheim, Germany) for reverse transcription. qPCR was conducted in a 10 μl reaction mixture containing 5 μl SSoAdvanced Universal SYBR Green Supermix (BioRad, Vienna, Austria), 1 μl template, 0.2 μl forward primer and 0.2 μl reverse primer (each 10 pmol/μl) and 3.6 μl dH_2_O in a StepOne Plus real-time PCR System (Applied Biosystems, Vienna, Austria). The following primer pairs were used: Irf5 (forward: 5´-AGAAGAGGAGGAAGAGGAAGA-3´; reverse: 5´- GCACAGGTTCTGTGATACTC-3´); Myo1f: forward: 5´- TAAGAGCACCAAGCCTACAC-3´; reverse: 5´- TGGTACCCCATTTCGATTCA-3´); Cotl1 (forward: 5´- GCGGATTACCAGCACTTCAT-3´; reverse: 5´- CAAAATTCTGGACCACCTCCT-3´); Psmb9 (forward: 5´- AGGACTTGTTAGCGCATCTC-3´; reverse: 5´- CATGGTTCCATACACCTGGC-3´); Gbp2 (forward: 5´- ACTTTGAGTCCAAGGAAGACA-3´; reverse: 5´- GCCTTAATCCGTTCCACTTC-3´); Tyrobp (forward: 5´- CAGGCCCAGAGTGACAATTAC-3´; reverse: 5´- CACAATCCCAGCCAGTACAC-3´); GAPDH (forward: 5´-CCGAGGGCCCACTAAAGG-3´; reverse: 5´-ATGGGAGTTGCTGTTGAAGTCA-3´). The reaction mixture was subjected to an initial denaturation step (30 seconds, 95°C), and then to 40 cycles of denaturation (15 seconds, 95°C) and annealing/extension (1 min, 60°C). ΔCT values were calculated using GAPDH as reference gene.

### Induction of ENMO and treatment with type I interferons

ENMO was established as described [18]. Essentially, Lewis rats were intraperitoneally injected with activated, MBP-specific T-cells on day 0, injected with 10mg NMO-IgG i.p. and 5x10^5^ units IFN-β (CHO-derived, U-Cytech, Utrecht, NL) or PBS i.v. on day 4. The clinical course of the disease was assessed using the following score: 0 = healthy; 0.5 = partial loss of tail tonus; 1 = complete loss of tail tonus; 2 = unsteady gait, hind limb weakness; 3 = bilateral hind limb paralysis. 12, 24, and 48 hours after the injection of NMO-IgG and IFN-β, the animals were killed by CO_2_ overdose. An additional batch of Lewis rats received 1x10^5^ units IFN-α1 (insect-cell derived; U-Cytech, Utrecht, NL) i.v. instead of IFN-β, and was killed 24 hours later by CO_2_. Then, the animals were perfused with 4% phosphate buffered paraformaldehyde (PFA). The spinal cords were dissected and immersed for another 18 hours in PFA. The PFA-fixed material was routinely embedded in paraffin and sectioned for immunohistochemical analysis.

### Immunohistochemistry

All stainings were done essentially as described [18] using the mouse monoclonal antibody ED1 (to stain macrophages and activated microglia; Serotec, Germany), rabbit polyclonal antibodies against CD3 (to stain T cells; NeoMarkers, Fremont, USA), rabbit polyclonal antibodies against AQP4 (to stain astrocytes; Sigma, Germany), rabbit polyclonal or mouse monoclonal antibodies against glial fibrillary acidic protein (GFAP; from Dako, Denmark, or NeoMarkers, respectively), anti-human immunoglobulin (biotinylated donkey; polyclonal; Amersham, UK), anti-complement C9 (rabbit polyclonal [21]), anti-Rab5c (goat polyclonal; Santa Cruz), anti-5-lipoxygenase (rabbit monoclonal; Cell signaling), and anti-Ptpn6 (rabbit polyclonal, Abnova). While the AQP4-specific antibodies could be used without antigen retrieval, the other antibodies required heat-mediated antigen retrieval by steaming the sections for 60 minutes in 50 μM EDTA pH 8.5 (ED1, antibodies against CD3, GFAP, Rab5c, 5-lipoxygenase and Ptpn6), or a treatment for 15 minutes at 37°C with 0.03% Proteinase Type XXIV (Sigma) (antibodies against human immunoglobulin and against complement C9).

### Quantitative analysis and statistical evaluation

The mean value of the number of antibody-reactive cells of EAE and ENMO animals was determined from 3 complete lumbar sections/animal, and the mean value of antibody-reactive cells and the area of AQP4 loss of I-IFN-treated ENMO animals were determined from 1 lumbar and 1 thoracic spinal cord/experimental animal, using a morphometric grid. The mean values of all animals/experimental group were then used to calculate medians and ranges. Statistical analysis was assessed by Mann-Whitney U test, using IBM SPSS Statistics ver. 21. P-values < 0.05 were considered as significant.

## Results

### Microarray analysis of ENMO spinal cords yields information about lesion pathogenesis

Our first round of gene expression studies revealed that 474 genes were upregulated in ENMO compared to any other experimental group. All genes with available GenBank accession numbers (n = 366) were then used as input for the database for annotation, visualization, and integrated discovery (DAVID, https://david.ncifcrf.gov/toolds/jsp) [22–24], to make GO term/pathway analysis. The functional annotation cluster 1, with an enrichment score of 2.66, revealed hits with the highest number of records in the GO term pathways “immune response” (25 records), “antigen processing and presentation” (11 records), “regulation of immune effector processes” (11 records), “positive regulation of immune responses” (12 records), and “defense response” (18 records) (S1 Table). These GO term pathways clearly indicated that the immune system plays an important role in the formation of lesions in the spinal cords of ENMO animals, but were not yet ideal for direct comparison with pathological findings. Therefore, we refined our analyses, and made searches using iHOP (http://www.ihop-net.org/) [25], published information about microarray data sets [26], and PubMed (http://www.ncbi.nlm.nih.gov/pubmed/) to ascribe differentially expressed genes to targets (i.e. astrocytes) and humoral (complement, cytokines) or cellular effectors of the immune system possibly involved in lesion pathogenesis.

**Fig 1:**
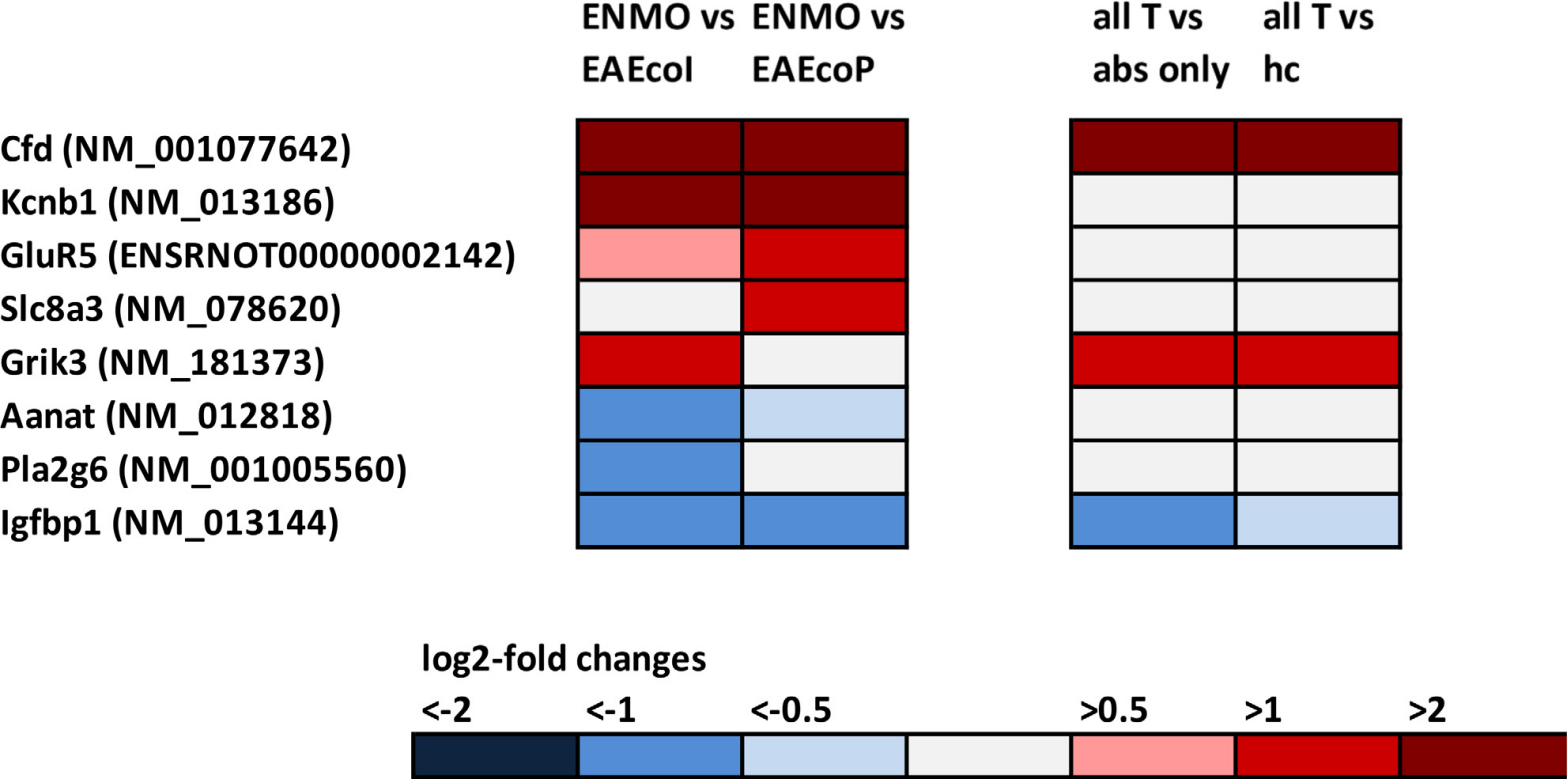
Footprints of genes suggesting astrocyte responses to excitotoxicity and injury in the spinal cord, as revealed by microarray analysis. In the first column of pairwise comparison of log_2_-fold changes in gene expression, mean values were compared between rats receiving T cells and NMO-IgG (ENMO, n = 5) and their counterparts receiving T cells and subcuvia as control IgG (EAE_coI_, n = 5) or T cells and PBS (EAE_coP_, n = 5). In the second column of pairwise comparison of log_2_-fold changes in gene expression, mean values were compared between a group containing all ENMO plus EAE_coI_ plus EAE_coP_ animals (n = 15, “all T”) and a group containing animals injected with antibodies only (“abs only” (5 animals with NMO-IgG plus 5 animals with subcuvia as control IgG) or containing healthy control animals only (“hc”, n = 3).

**Fig 2:**
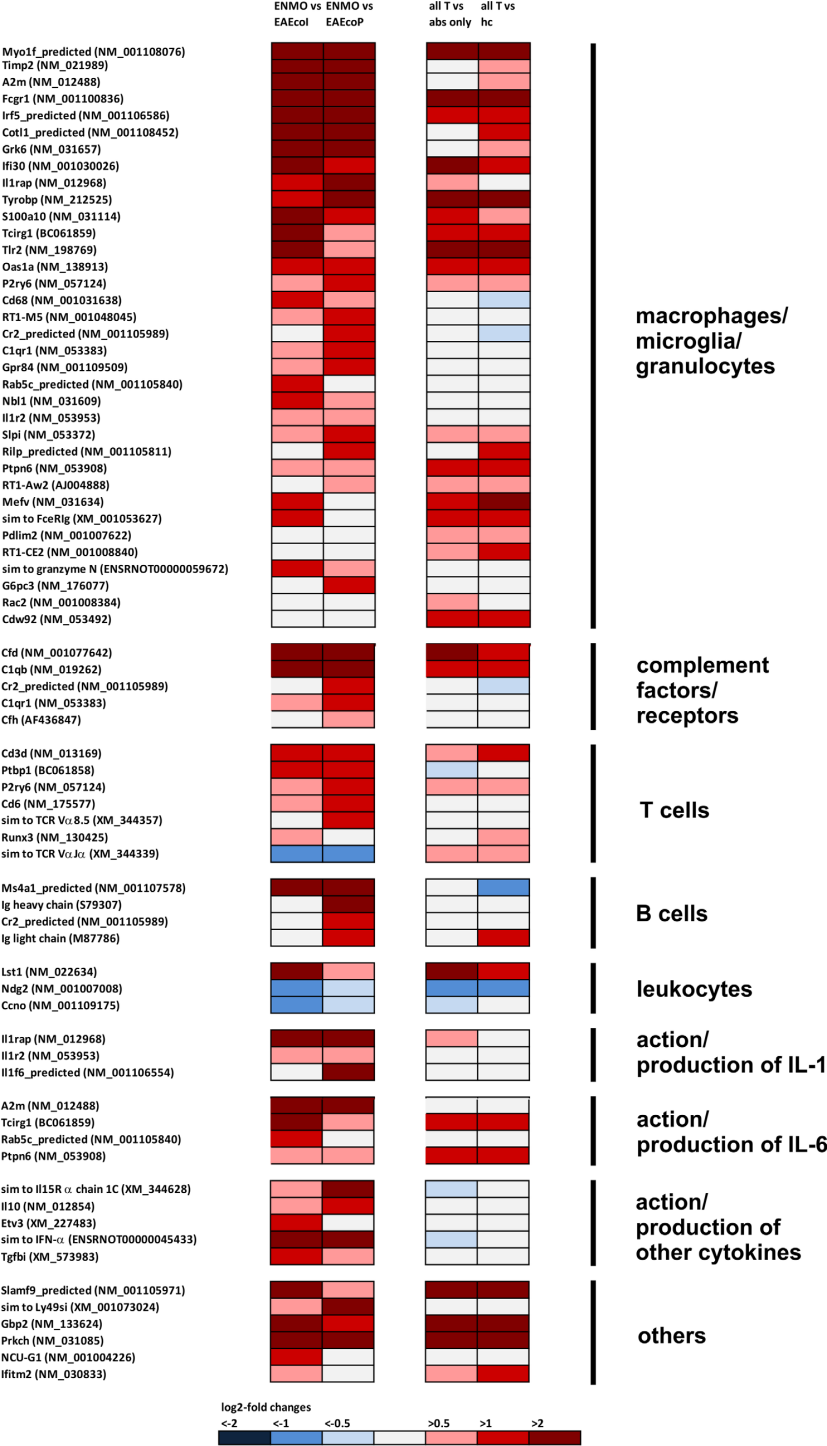
Footprints of inflammatory processes in the spinal cord, as revealed by microarray analysis. In the first column of pairwise comparison of log_2_-fold changes in gene expression, mean values were compared between rats receiving T cells and NMO-IgG (ENMO, n = 5) and their counterparts receiving T cells and subcuvia as control IgG (EAE_coI_, n = 5) or T cells and PBS (EAE_coP_, n = 5). In the second column of pairwise comparison of log_2_-fold changes in gene expression, mean values were compared between a group containing all ENMO plus EAE_coI_ plus EAE_coP_ animals (n = 15, “all T”) and a group containing animals injected with antibodies only (“abs only” (5 animals with NMO-IgG plus 5 animals with subcuvia as control IgG) or containing healthy control animals only (“hc”, n = 3).

We found differential expression of 8 genes suggesting astrocyte responses to excitotoxicity and injury (Fig 1, Table 1), and upregulation of genes involved in inflammatory processes: 35 genes indicative of presence and/or activation of granulocytes, microglia, and macrophages; 5 genes of the complement pathway; 14 genes revealing the presence of T and B lymphocytes; and 10 genes encoding interleukins/interleukin receptors or suggesting the action/production of these molecules (Fig 2).

**Table 1:** Differentially expressed astrocyte-related genes in spinal cords of Lewis rats with experimental neuromyelitis optica.

Target Id	fc ENMO/ EAE	fcall T / all non-T	Gene symbol	major function	Ref.
NM_001077642	11.8	4.3	Cfd	complement factor D (adipsin); alternative complement pathway; found in astroglioma	[42]
NM_013186	5.7	1.0	Kcnb1	potassium voltage gated channel, Shab-related subfamily, member 1; Kv2.1; on neurons apposed to astrocytic processes	[43]
ENSRNOT00000002142	1.8	1.2	GluR5	Glutamate receptor, ionotropic kainate 1 precursor (Glutamate receptor 5) (GluR-5) (GluR5); = Grik1; specifically expressed at perivascular astrocytic processes;	[44]
NM_078620	1.7	1.1	Slc8a3	solute carrier family 8 (sodium/calcium exchanger), member 3; highly expressed in astrocytes in response to glutamate-induced excitotoxicity	[45]
NM_181373	1.6	2.2	Grik3	glutamate receptor, ionotropic, kainate 3 (Grik3), transcript variant 2; = GluR7; throughout the astrocyte; not limited to vascular profiles	[44]
NM_012818	0.5	1.3	Aanat	arylalkylamine N-acetyltransferase; in astrocytes after transient ischemia	[46]
NM_001005560	0.4	0.9	Pla2g6	phospholipase A2, group VI; = iPLA2; increased expression in astrocytes leads to augmented Ca2+ signaling in response to purinergic ATP signaling. Silencing associated with amplified prostaglandin release by astrocytes.	[47,48]
NM_013144	0.3	0.5	Igfbp1	insulin-like growth factor binding protein 1; leads to, reduced astrocytic response to injury upon overexpression; found in hypertrophic astrocytes of MS lesions;	[49,50]

fold changes (fc) above 1 indicate an upregulation in gene expression, fc below 1 indicate a downregulation.

EAE = mean value of EAE_coI_ and EAE_coP_; all T = mean value of all T cell mediated diseases (i.e. ENMO, EAE_coI_, EAE_coP_)

all non-T = mean value of all non-inflammatory controls (i.e. healthy control animals, animals injected with NMO-IgG only, animals injected with control IgG only).

Cumulatively, all the identified changes in gene expression are fully in line with the pathological changes observed in ENMO animals, which are T-cell mediated CNS inflammation and astrocyte-destruction by complement-mediated cytotoxicity [1,18] and antibody-dependent cellular cytotoxicity [27,28] executed by activated microglia/macrophages and neutrophils. A similar accordance between tissue changes and gene expression data had been observed before by Inglis and colleagues, who analyzed spinal cords of Lewis rats at the peak of active EAE [29]. This suggested that the microarray data on our FFPE material faithfully reflect the tissue changes observed in histology [18].

We also detected an IL-6 signature, as evidenced by the up-regulation of A2m, Tcirg1, Rab5c_predicted, and Ptpn6 (Fig 2). This was remarkable since IL-6 signaling is known to play an important role in NMO [30,31]. Noteworthy, we also found an upregulation of ENSRNOT00000045433 (= “similar to IFN-α”; Fig 2, S2 Table).

To further verify the expression of some of the upregulated gene products in ENMO vs EAE, we performed immunohistochemical analysis, concentrating on Ptpn6, Rab5c, and 5-lipoxygenase (S2 Table).

**Fig 3:**
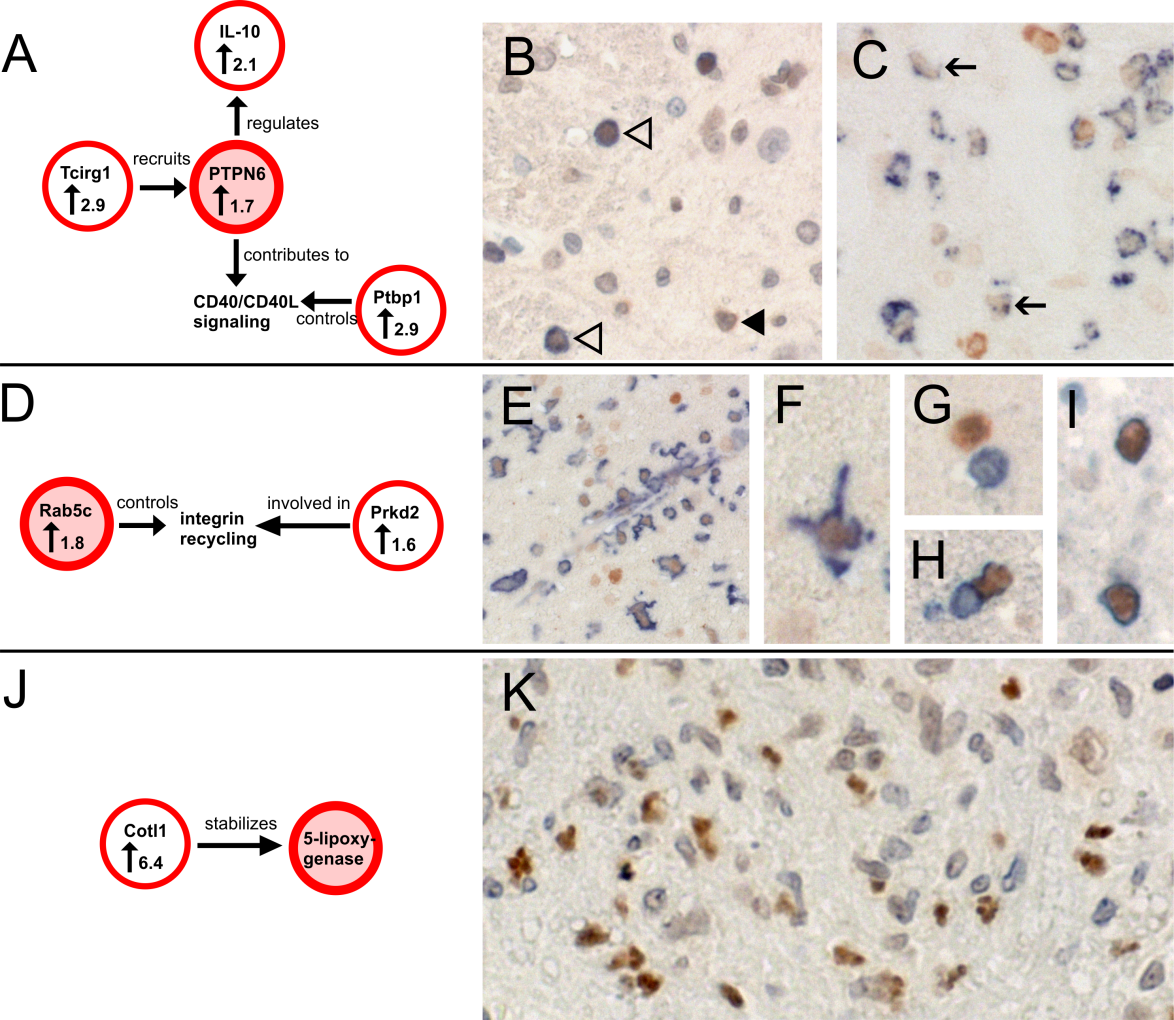
Histological confirmation of the expression and cellular sources of key molecules identified by microarray analysis. (A) Interconnection of Ptpn6 with other molecules differentially upregulated (↑, fold change) in ENMO compared to EAE. Ptpn6 is recruited by Tcirg1 [33,34], regulates the production of IL-10 [35], and contributes to CD40 signaling reciprocity [36]. A critical molecule for turnover and subcellular distribution of CD40L is Ptbp1 [37]. Hence, confirmation of Ptpn6 expression supports gene expression data of three additional differentially expressed genes. (B) Spinal cord section of a Lewis rat with ENMO reacted with antibodies against CD3 (blue surface staining) and Ptpn6 (brown). The section was faintly counterstained with hematoxylin to reveal nuclei in blue. Shown here is Ptpn6 expression in CD3^+^ T cells (white arrow heads) and in neutrophils with lobulated nuclei (black arrow head). (C) Spinal cord section of a Lewis rat with ENMO reacted with the ED1 antibody (blue) and Ptpn6 (brown). In ED1^+^ activated microglial cells/macrophages, Ptpn6 expression is low (black arrow). (D) Interconnection of Rab5c, which regulates the endocytic pathway and controls the rates of integrin internalization and recycling [38] with Prkd2, a molecule involved in β1 integrin recycling [39]. Both molecules are differentially upregulated (↑, fold change) in ENMO compared to EAE. (E-I) Spinal cord section of a Lewis rat with ENMO reacted with antibodies against Ptpn6 (brown) and Iba 1 (blue) to show the expression of Ptpn6 in microglia (E,F), CD3 (blue) to show the absence of Ptpn6 expression in CD3^+^ T cells (G), and W3/13 (blue) to show the expression of Ptpn6 in neutrophils (H,I). (J) 5-lipoxygenase is stabilized by Cotl1 [40,41], a molecule found 6.4-fold upregulated (↑, fold change) in ENMO compared to EAE (S1 Table). (K) Spinal cord section of a Lewis rat with ENMO reacted with antibodies against 5-lipoxygenase (brown). The section was faintly counterstained with hematoxylin to reveal nuclei in blue. 5-lipoxygenase is localized to the lobulated nuclei of neutrophils.

**Fig 4:**
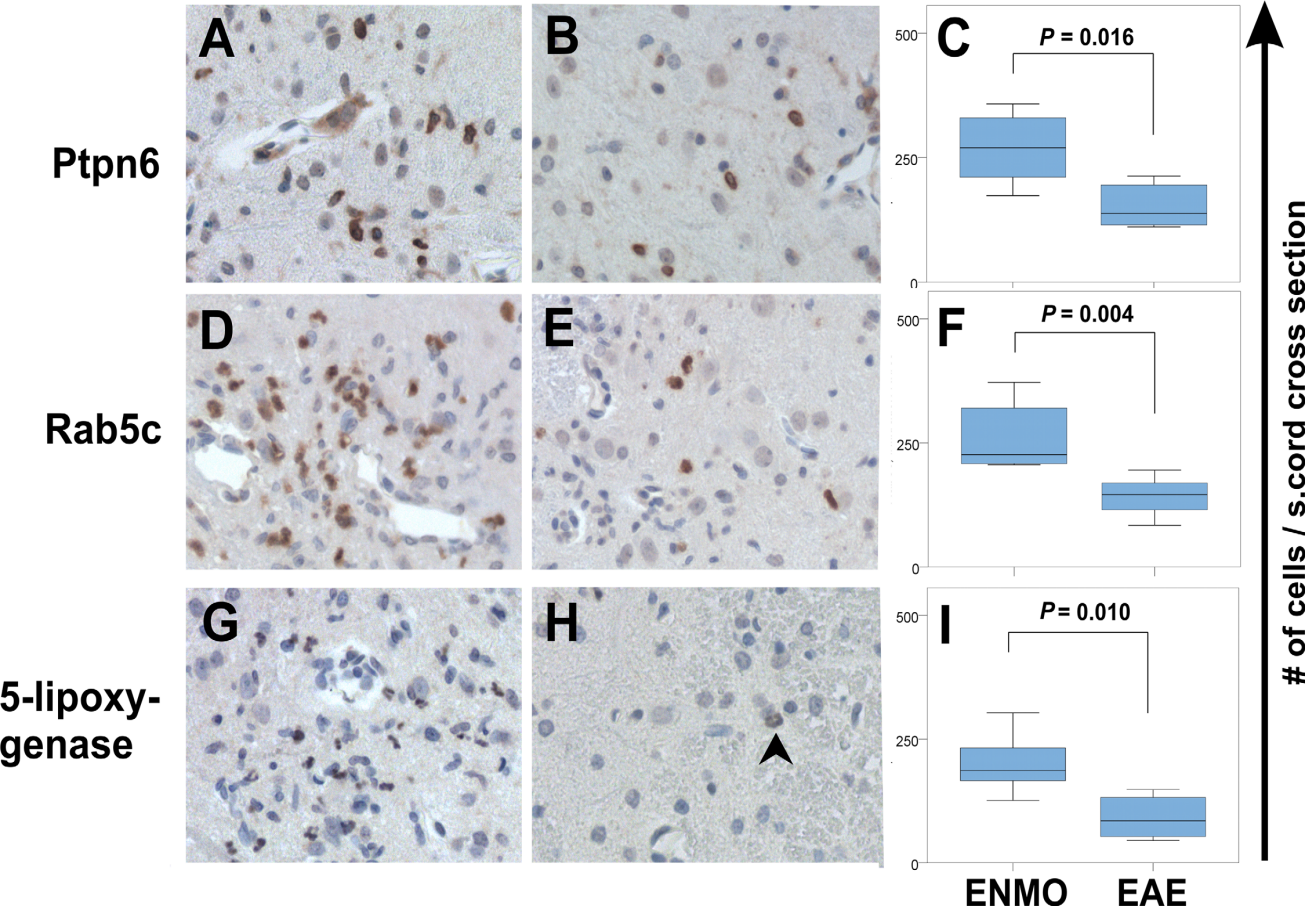
Confirmation by immunohistochemistry of differential expression of Ptpn6, Rab5c and 5-lipoxygenase in ENMO and EAE. Shown here are cross sections of spinal cords from animals with ENMO (A, D, G) and EAE_coI_, (B, E, H) reacted with antibodies against Ptpn6 (A, B), Rab5c (D,E) and 5-lipoxygenase (G,H). Reaction products are brown. Counterstaining was done with hematoxylin to reveal nuclei (blue). Statistically significant differences in the number of Ptpn6- (C), Rab5c- (F), and 5-lipoxygenase- (I) positive cells / spinal cord sections between ENMO and EAE_coI_ are seen (Mann-Whitney U-test). Shown here are medians (range). The arrow in (H) points to a weakly stained nucleus of a neutrophil.

Staining of spinal cords with Ptpn6-specific antibodies revealed the expression of this molecule in activated microglia/macrophages, some neutrophils and T cells (Fig 3A–3C) with higher numbers of these cells in ENMO than in EAE (Fig 4A–4C).

Stainings of spinal cords with anti-Rab5c antibodies shows expression of Rab5c in microglia and neutrophils (Fig 3D–3I). The number of Rab5c^+^ cells is higher in ENMO than in EAE (Fig 4D–4F)

Stainings of spinal cords with anti-5-lipoxygenase antibodies yielded higher numbers of brown, lobulated nuclei in ENMO spinal cords than in their EAE counterparts, which is in line with the location of 5-lipoxygenase in the nuclear envelope of activated neutrophils [32] (Fig 3J and 3K), and with the higher numbers of these cells in ENMO compared to EAE [18] (Fig 4G–4I).

**Fig 5:**
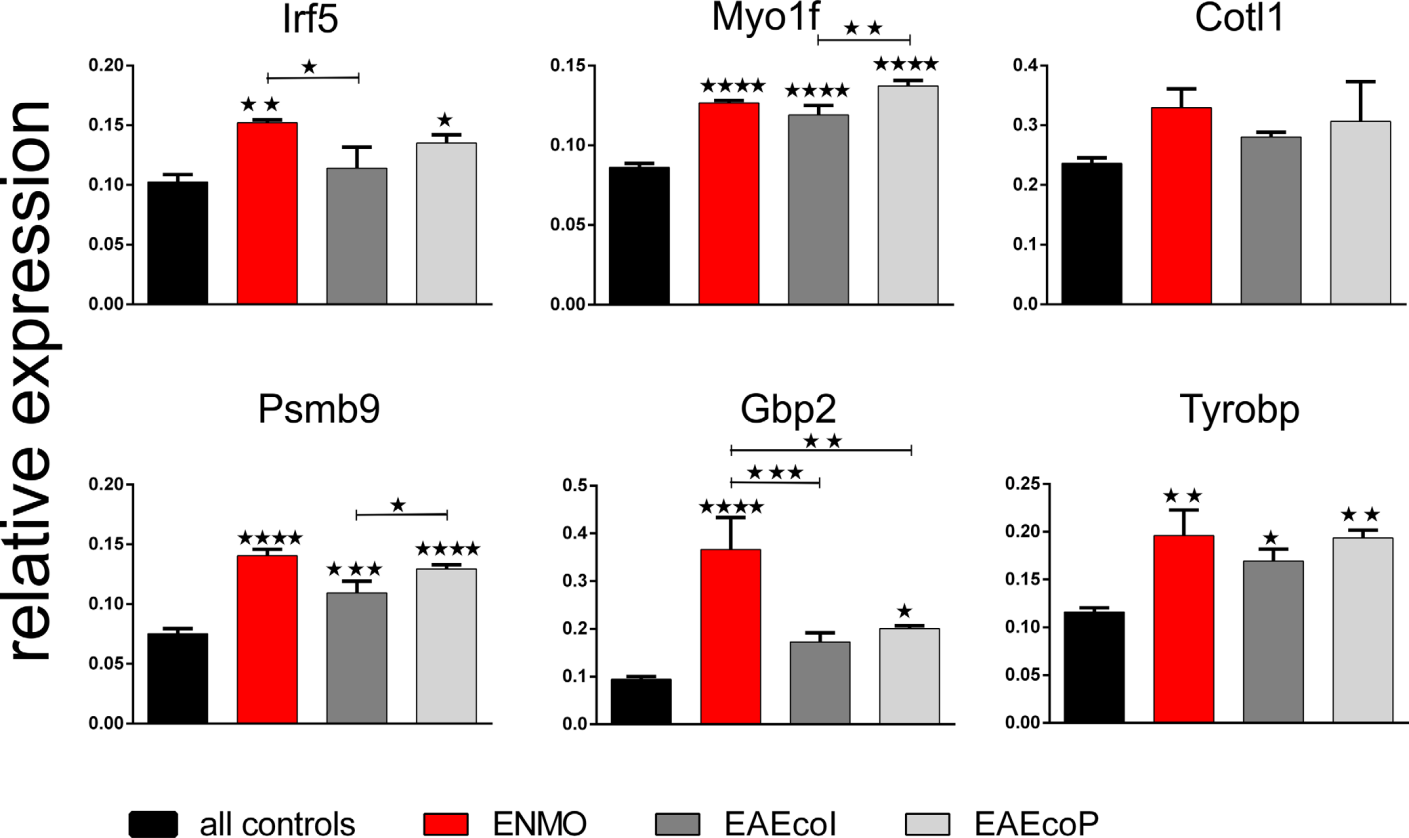
Confirmation of differentially expressed genes by qPCR. Shown here are the mean relative expression values (+/-SEM) of different gene products in relation to the house keeping gene glyceraldehyde-3-phosphate-dehydrogenase (GAPDH) of rats receiving T cells and NMO-IgG (ENMO, n = 3), T cells and subcuvia as control IgG (EAE_coI_, n = 3) or T cells and PBS (EAE_coP_, n = 3) in comparison to “all controls” (mean value of rats injected with NMO-IgG only (n = 2), subcuvia as control IgG only (n = 3) and PBS only (n = 3). Unless otherwise indicated, statistically significant differences of the experimental groups are calculated in relation to “all controls”. (*p<0.05, **p<0.01, ***p<0.001, ****p<0.0001, one-way ANOVA with Bonferroni multiple comparisons test).

In addition to histological verification, we also verified some of the upregulated gene products by qPCR. Although the cDNA for this experiment derived from fresh tissue and had not been amplified before, as was the case for the FFPE material used for microarray analysis, we could confirm statistically significant higher levels of gene expression for Irf5, Myo1f, Psmb9, Gbp2 and Tyrobp, and a trend for higher expression of Cotl1 in ENMO vs all controls (= NMO-IgG, subcuvia, PBS), and we could also confirm statistically significant higher levels of gene expression for Irf5 compared to EAE_coI_, and for Gbp2 compared to EAE_coI_ and to EAE_coP_. There was a trend for higher expression levels of Cotl1, Psmb9 and Tyrobp compared to EAE_coI_ and to EAE_coP_ (Fig 5). Although Myo1f was expressed at higher levels in in ENMO vs all controls, it was–in contrast to the microarray data–not expressed at significantly higher levels in ENMO vs EAE_coI_ or EAE_coP_. The most likely reason for this discrepancy is a non-linear amplification of Myo1f transcripts during cDNA amplification of the FFPE-derived material prior to microarray analysis.

### Microarray analysis of ENMO spinal cords reveals footprints of the action/production of I-IFN

**Fig 6:**
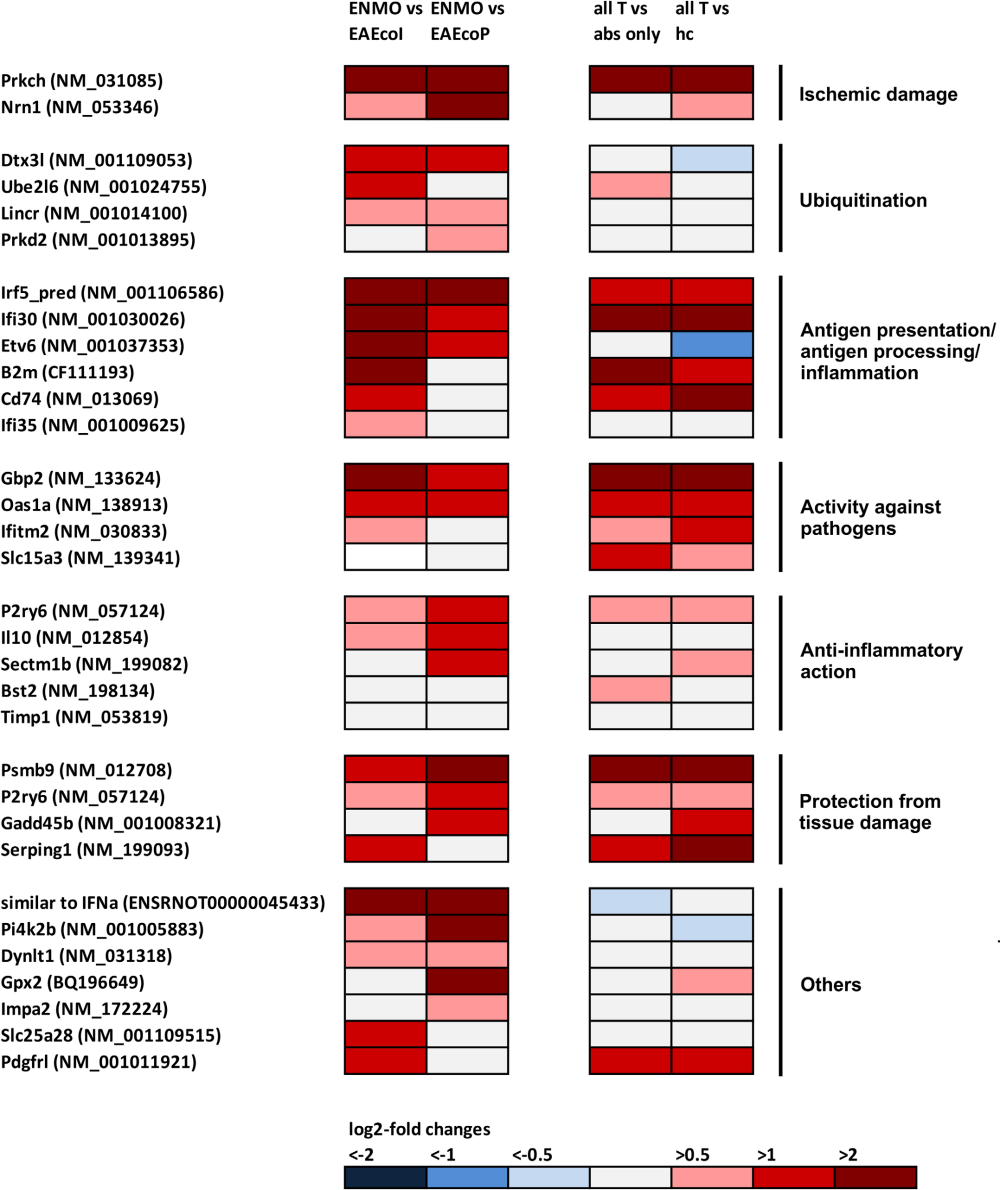
Footprints of the action/production of type I interferons in ENMO and EAE, as revealed by microarray analysis. In the first column of pairwise comparison of log_2_-fold changes in gene expression, mean values were compared between rats receiving T cells and NMO-IgG (ENMO, n = 5) and their counterparts receiving T cells and subcuvia as control IgG (EAE_coI_, n = 5) or T cells and PBS (EAE_coP_, n = 5). In the second column of pairwise comparison of log_2_-fold changes in gene expression, mean values were compared between a group containing all ENMO plus EAE_coI_ plus EAE_coP_ animals (n = 15, “all T”) and a group containing animals injected with antibodies only (“abs only” (5 animals with NMO-IgG plus 5 animals with subcuvia as control IgG) or containing healthy control animals only (“hc”, n = 3). The differentially expressed genes shown here belong to 7 different, large groups, i.e. to ischemic damage, ubiquitination, antigen presentation/antigen processing/inflammation, activity against pathogens, anti-inflammatory action, protection from tissue damage, and unknown function (“others”). In experimental autoimmune neuromyelitis optica (ENMO), 31 differentially expressed genes are found. 19/32 differentially expressed genes were already upregulated in all T cell-induced CNS inflammations compared to all other non-inflammatory controls.

Since we have identified ENSRNOT00000045433 (= “similar to IFN-α”) as up-regulated gene in ENMO spinal cords, and since NMO patients have an increased I-IFN signature in the serum [51,52], we next searched whether our gene expression studies by microarrays hit upon any other I-IFN-stimulated gene (ISG) in the ENMO spinal cords. For this purpose, we used a list of 387 human/chimpanzee ISGs compiled by Schoggins and colleagues [26] after screening data sets from 10 different publications on microarrays from various I-IFN-treated cells or tissues [53–62], and also made additional literature searches [63–65]. We found 31 ISGs among the differentially expressed genes in ENMO spinal cords (Fig 6, Table 2), most noteworthy interferon gamma inducible protein 30 (Ifi30, also known as gamma-interferon-inducible lysosomal thiol reductase (GILT)), which counts among the top 20 upregulated genes in NMO lesions [66]. Since GO Term pathway analysis only insufficiently assigned these genes to specific groups, we used PubMed searches to cluster them according to their possible involvement in ischemic damage (2), ubiquitination (4), antigen processing/presentation and inflammation (6), activity against pathogens (4), anti-inflammatory action (5), protection from tissue damage (4), and others (7) (Fig 6, S3 and S4 Tables). Cumulatively, these findings revealed that ENMO rats have a clear type I-IFN signature in the spinal cord.

**Table 2:** Footprints of the action/production of type I interferons in spinal cords of Lewis rats with experimental neuromyelitis optica.

Target Id	fc ENMO / EAE	fc all T / all non-T	Gene symbol	major function	Ref.
**NM_031085**	**432.4**	**72.4**	**Prkch**	protein kinase C eta; down-regulated through immune responses; associated with increased risk of rheumatoid arthritis, ischemic stroke and cerebral hemorrhage	[76,77]
**NM_001106586**	**6.8**	**3.1**	**Irf5_predicted**	interferon regulatory factor 5 (predicted); highly expressed in M1 macrophages; promotes polarization of inflammatory macrophages and T_H_1-T_H_17 responses.	[78]
NM_001030026	5.6	5.1	Ifi30	interferon gamma inducible protein 30; = Gamma-interferon-inducible lysosomal thiol reductase (GILT); involved in antigen-processing by antigen presenting cells; found among top 20 upregulated genes in NMO lesions	[66,79]
**ENSRNOT00000045433**	**5.0**	**0.7**	**ENSRNOT00000045433**	**„Similar to interferon-α “**	
NM_012708	3.6	21.5	Psmb9	Component of immunoproteasome; protect cell viability under conditions of IFN-induced oxidative stress; critical for removal of oxidized proteins	[80,81]
NM_001037353	3.2	0.7	Etv6	ets variant gene 6 (TEL oncogene); represses Stat3, which is a transcription factor needed for the antiproliferative effects caused by cytokines like IL-6	[82]
NM_133624	3.0	83.7	GBP2	guanylate nucleotide binding protein 2; inhibits cell spreading; role in resistance to intracellular pathogens	[83,84]
NM_001005883	2.8	0.7	Pi4K2B	Phosphatidylinositol 4-kinase type 2-beta	[25]
**NM_138913**	**2.6**	**3.6**	**Oas1a**	2'-5' oligoadenylate synthetase 1A; in antiviral signaling cascade	[64]
NM_001109053	2.4	0.7	Dtx3l	Deltex 3-like; E3 ubiquitin-protein ligase	[85]
NM_053346	2.4	1.0	Nrn1	Neuritin; induced by hypoxia; hypoxic perinecrotic marker	[86]
NM_057124	2.3	1.6	P2ry6	pyrimidinergic receptor P2Y, G-protein coupled, 6; in T cells and macrophages; inhibits activation of effector T cells; in astrocytes: activation prevents TNF-α-induced apoptosis in astrocytes;	[87–89]
**NM_012854**	**2.1**	**0.8**	**Il10**	**Interleukin 10; antiinflammatory action**	[25]
**NM_001008321**	**2.0**	**1.4**	**Gadd45b**	**Growth arrest and DNA-damage-inducible, beta; regulates cell growth, differentiation and cell death following cellular exposure to DNA damage and TGF-β**	**[90]**
NM_199093	2.0	3.3	Serpin G1	C1 esterase inhibitor; prevents complement factor C1 autoactivation in the fluid phase and prevents initiation of classical-pathway activation on antigen–antibody complexes when the antibody has low antigen affinity or interacts weakly with C1q.	[91–93]
CF111193	2.0	4.9	B2m	Beta-2-microglobulin; antigen presentation via MHC class I	[25]
NM_001024755	1.9	1.4	Ube2l6	ubiquitin-conjugating enzyme E2L 6;	[25]
NM_001014100	1.7	1.1	Lincr	E3 ubiquitin-protein ligase NEURL3;	[25]
NM_031318	1.7	0.8	Dynlt1	Dynein light chain TcTex-type 1; upon phosphorylation, it regulates microtubules and mitochondria, leads to their stabilization, and contributes to cellular hypoxic tolerance	[94]
NM_199082	1.7	2.8	Sectm1b	Secreted and transmembrane 1B; inhibitory to T cell receptor-mediated T cell activation	[95]
BQ196649	1.7	1.3	Gpx2	glutathione peroxidase 2; mostly described in the context of intestinal inflammation	[25]
NM_172224	1.6	1.0	Impa2	inositol (myo)-1(or 4)-monophosphatase 2; mostly described in context of bipolar disorders	[25]
NM_001109514	1.6	1.2	Slc25a28	Mitoferrin-2; Mitochondrial iron transporter	[25]
NM_013069	1.6	2.8	CD74	Invariant chain functioning as MHC class II chaperone; a chondroitin-sulfate modified CD74 is expressed on the surface of antigen-presenting cells as part of the CD44-CD74 receptor complex. This complex found both in macrophages/monocytes and B cells and is needed for the binding of macrophage migration inhibitory factor (MIF). In macrophages/monocytes, this leads to the subsequent activation of these cells for optimal expression of TNF, IL-1, and prostaglandin E2, and for enhancing phagocytosis; In B cells, it causes proliferation/survival and results in maintaining a mature B cell population	[96]
NM_001011921	1.6	3.1	PDGFRL	Platelet-derived growth factor receptor-like protein	[25]
NM_001013895	1.6	1.3	Prkd2	Protein kinase D2; involved in β1 integrin recycling upon activation of Rab5c; required for ligand-inducible stimulation of IFNAR1 ubiquitination and endocytosis; many additional functions	[25,39,97]
**NM_030833**	**1.4**	**2.2**	**Ifitm2**	interferon induced transmembrane protein 2; anti-viral	[26]
NM_001009625	1.4	1.1	Ifi35	Negatively regulates antiviral signaling	[98]
NM_139341	1.2	1.9	Slc15a3	Endo-lysosomal peptide transporter; preferentially expressed by dendritic cells after activation of Toll-like receptors; mediates egress from peptides into the cytoplasm for pathogen sensing by NOD2 (nucleotide-binding oligomerization domain containing 2); Activation of NOD2 results in the transcription of genes encoding chemokines, cytokines, antimicrobial peptides, and type I interferons	[99,100]
NM_198134	1.2	1.4	Bst2	Bone marrow stromal cell antigen 2 (CD317); readily induced by type I interferons; strongly inhibits production of IFN and proinflammatory cytokines by plasmacytoid dendritic cells	[101]
NM_001037353	1.2	1.2	Timp1	Tissue inhibitor of metalloproteinases; attenuates blood-brain barrier permeability; regulates access of CD4+ T cells into the CNS parenchyma	[102,103]

Type I interferon stimulated genes were identified using a list of 387 type I interferon stimulated human/chimpanzee genes compiled by Schoggins and colleagues [26] after screening data sets from 10 different publications on microarrays from various type I IFN-treated cells or tissues [53–62], and additional literature searches (bold) [63–65].

Fold changes (fc) > 1 indicate an upregulation in gene expression, fc < 1 indicate a downregulation all T = mean value of all T cell mediated diseases (i.e. ENMO, EAE_coI_, EAE_coP_)

EAE = mean value of EAE_coI_ and EAE_coP_; all non-T = mean value of all non-inflammatory controls (i.e. healthy control animals, animals injected with NMO-IgG only, animals injected with control IgG only)

Most of the ISGs were already upregulated in EAE (Fig 6, S3 Table, S4 Table), but were further increased in ENMO (Fig 6, Table 2). The upregulation of ISGs in ENMO compared to EAE suggests that they either influence the formation of inflammatory spinal cord lesions provoked by the presence of antibodies and granulocytes in ENMO [18,67], or that they are specifically triggered by this process. These findings raised important questions:

Is the size of astrocyte-destructive lesions seen in ENMO limited by ISGs, as suggested by the observation of a protective role of I-IFN signaling in EAE [15]? Is their size promoted by the action of these genes, as suggested by the formation of larger astrocyte-destructive lesions after intra-cerebral injection of NMO-IgG and complement in I-IFN receptor (IFNAR) sufficient animals than in their knock-out counterparts [16]? Or is the action of I-IFNs neutral, as suggested from a lack of potentiation of lesions in spinal cord slice cultures exposed to complement and NMO-IgG for 72 hours after a 24-hour pretreatment with IFN-β- [17]?

To specifically address these questions, we could not applicate I-IFN in an active ENMO model induced by immunization with AQP4 in complete Freund´s adjuvans since I-IFN interferes with T cell–dendritic cell interactions in lymph nodes and thus skews the activation and expansion of T cell subsets [68,69].

Instead, we initiated passive ENMO by transfer of CNS antigen-specific T cells and transfer of both NMO-IgG and I-IFN or vehicle at the time when first clinical symptoms indicated an open blood-brain barrier. Under these conditions, I-IFN could enter the CNS not only unspecifically and passively [70–73], but also in the correct temporal and spatial context of lesion formation. In such a scenario, the actions of I-IFNs in lymph nodes during the priming phase of immune responses could be neglected, and the observed effects would only result from an I-IFN effect on or at the blood brain barrier affecting leukocyte trafficking, and from the amplification of the local I-IFN responses by the peripherally administered I-IFNs, since “even twofold changes in IFN levels can result in sixtyfold changes in ISG levels” [74,75]. We reasoned, that under such conditions, beneficial or detrimental effects of the ISGs should become clearly visible.

### ENMO in the presence or absence of the administration of type I interferons

**Fig 7:**
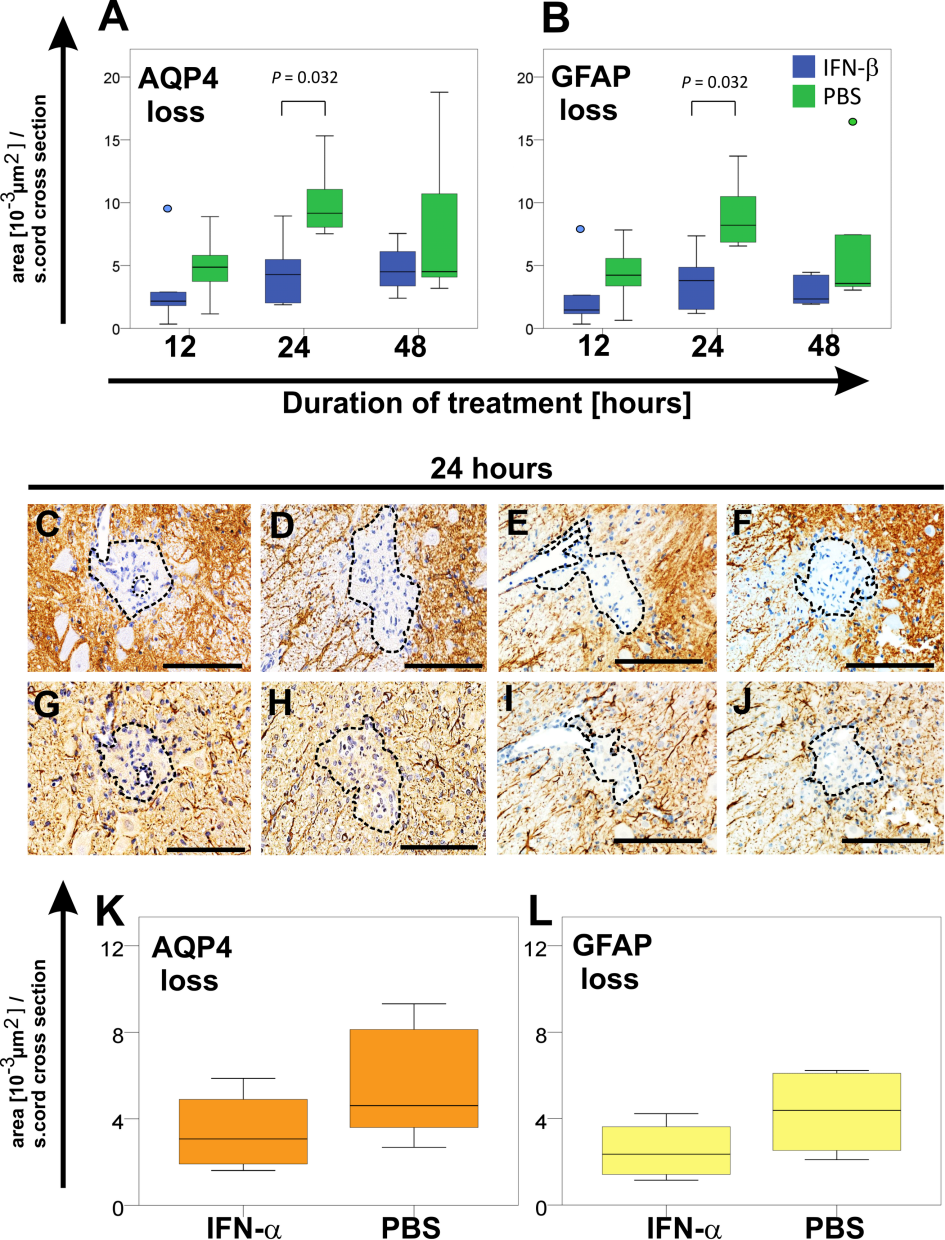
Differences in tissue damage between type I interferon-treated animals with ENMO. Size determinations of lesions with loss of AQP4 (A) or GFAP (B) reactivity in spinal cord sections of ENMO animals treated with IFN-β (blue) or PBS as vehicle control (green) for 12, 24, or 48 hours. Shown here are median and range of 5 animals per group. Differences between IFN-β and PBS-treated animals were significant after a 24-hour treatment with IFN-β (p = 0.032, Mann-Whitney U test; The blue and green dots indicate outliers). Shown in C-J are representative spinal cord sections reacted with antibodies against AQP4 (C-F, brown) or GFAP (G-J, brown) of animals treated for 24 hours with IFN-β (C,G) or PBS (D, H), and with IFN-α (E,I) or PBS (F,J). Counterstaining was done with hematoxylin to reveal nuclei (blue).

In a first set of experiments, we treated the ENMO animals with IFN-β or PBS. At the day of sacrifice, we found comparable clinical scores and NMO-IgG titers, which was ENMO median score 1 with a median antibody titer of 1:80 (range 1:40–1:80; animals killed 12 and 24 hours after IFN-β injection), and ENMO score 2 with a median antibody titer of 1:40 (range 1:20–1:40; animals killed 48 hours after IFN-β injection). We also observed the presence of inflammatory astrocyte-destructive lesions characteristic for ENMO in both types of animals. At all treatment times analyzed, spinal cord lesions with AQP4 loss and GFAP loss were smaller, and contained less CD3^+^ T cells, ED1^+^ macrophages/activated microglia and 5-LO^+^, activated neutrophils in IFN-β treated ENMO animals than in their PBS-treated counterparts. These differences reached significance for a treatment duration of 24 hours (Fig 7, Table 3).

**Table 3:** Comparison of immunohistochemical findings in ENMO animals treated for 12, 24, or 48 hours with interferon-beta (IFN-β) or phosphate-buffered saline (PBS; vehicle control).

12 hours			24 hours		48 hours	
	IFN-β	PBS	IFN-β	PBS	IFN-β	PBS
# lesions with AQP4 loss	1.1(0.3–2.4)	1.7(0.8–1.9)	1.2(0.7–1.6)	1.4(1.2–1.7)	1.0(0.7–1.4)	0.9(0.8–1.8)
# CD3^+^ cells	353 (328–489)	446(291–554)	298**(271–340)	540 **(440–642)	460 (393–517)	519 (308–589)
# ED1^+^ cells	982 (899–1142)	1004 (974–1108)	1024** (952–1184)	1584**(1456–1696)	868 (736–1344)	1048 (832–1460)
# 5-LO^+^ cells	179 (132–286)	241 (197–339)	120** (98–134)	238** (204–304)	94 (43–130)	112 (53–174)

5 animals/group were analyzed, and all data shown represent numbers (#) / complete spinal cord section expressed as median (range).

CD3^+^ cells represent T lymphocytes; ED1^+^ cells represent activated microglia/macrophages; 5-LO^+^ cells represent activated neutrophils.

** indicates statistically significant differences between the IFN-β and PBS treated animals with experimental neuromyelitis optica (p<0.01, Mann-Whitney U test).

Although IFN-β and IFN-α act through IFNAR, the functional outcome might be different (for review see [74]), either due to differences in the affinity of IFNAR for these molecules [104], or due to differences in the stability of IFNAR with its ligands [105,106]. Therefore, we made an additional experiment and treated the ENMO animals with IFN-α1 or PBS. When we sacrificed the animals 24 hours later, they had comparable ENMO scores (1.3 vs 1.5, p = 0.777), comparable antibody titers (median 1:240 vs 1:320, p = 0.091), and ENMO-typical lesions. As seen before with IFN-β treatment, lesions with AQP4 loss (Fig 7) and GFAP loss (Table 4) were smaller upon treatment with IFN-α1, although these differences did not reach significance.

**Table 4:** Comparison of immunohistochemical findings in ENMO animals treated for 24 hours with interferon-alpha (IFN-α) or phosphate-buffered saline (PBS; vehicle control).

	IFN-α	PBS
# lesions with AQP4 loss	1.5 (0.9–1.9)	1.9 (1.5–2.5)
# CD3^+^ cells	514 (505–660)	652 (587–692)
# ED1^+^ cells	1096 (918–1394)	1392 (1096–1843)
# 5-LO^+^ cells	127 (92–148)	205 (98–212)

4 and 5 animals/group were analyzed in the IFN-α and PBS-treated groups, respectively. The data shown represent numbers (#) / complete spinal cord section expressed as median (range). CD3^+^ cells represent T lymphocytes; ED1^+^ cells represent activated microglia/macrophages; 5-LO^+^ cells represent activated neutrophils. The differences between the different treatment groups were not significant (all p > 0.05, Mann-Whitney U test).

Taken together, treatment of ENMO animals with I-IFN under conditions of an open blood-brain barrier was clearly beneficial for the animals.

Median size and range of lesions with AQP4 loss (K) or GFAP loss (L) were also determined after a 24-hour treatment with IFN-α (4 rats) or PBS (5 rats). There was a trend towards smaller lesions resulting from IFN-α treatment, but did not reach significance (p = 0.221, Mann-Whitney U test).

## Discussion

We report here that Lewis rats with ENMO have a clear I-IFN signature in their spinal cords, as evident from the expression of ENRSRNOT00000045433 (“similar to interferon-α”), and also from the expression of ISGs. Although many of these gene products are already upregulated in EAE compared to non-inflammatory controls, the I-IFN signature is clearly more pronounced in ENMO than in EAE. Short-term I-IFN treatment of ENMO rats with an open blood-brain barrier limited the extent of tissue damage.

In the intact CNS parenchyma, I-IFN levels are extremely low, since plasmacytoid dendritic cells, the main IFN-α-producing cells [74,107] are absent, since astrocytes and neurons synthesize I-IFNs only after engagement of their toll-like receptors 3 in response to viral stimulation [108,109], and since oligodendrocytes seem to be unable to produce I-IFNs at all [74]. However, there are a number of reports suggesting that peripheral I-IFN is able to access the CNS [70–73].

In the inflamed CNS, I-IFNs are produced by infiltrating myeloid cells (dendritic cells, macrophages), and by cells with microglial morphology [110,111], while responses to I-IFN can be mounted by many types of cells expressing the I-IFN receptor (IFNAR; [112]), e.g. by infiltrating macrophages [113], plasmacytoid dendritic cells [114–116], neutrophils [117], microglia [118], T cells [119], and astrocytes [120]. In spite of the widespread expression of IFNARs, IFNAR-signaling in EAE and ENMO seems to predominantly affect myeloid cells, since many of the ISGs identified by our microarray analysis are either produced by or act on macrophages/activated microglia and neutrophils (Table 2).

We found that the expression of ISGs is higher in ENMO spinal cords than in their EAE counterparts, which is in line with the higher numbers of activated microglia/macrophages in the inflamed ENMO spinal cords [18], and with the induction of a pro-inflammatory, monocyte recruiting phenotype in astrocytes upon binding of NMO-IgG to AQP4 on their cell surface [121]. Moreover, when we further enhance local I-IFN levels by intraveneous injections of I-IFN at the onset of lesion formation, the amount of tissue damage caused by NMO-IgG was clearly reduced. It could be argued that we see less tissue damage due to an enhancement of activation-induced apoptosis by I-IFN. However, this is unlikely to be the cause, since this would affect T_H_17 cells much more than T_H_1 cells [122], the T cell subset used to induce ENMO [28][123]. Our findings clearly corroborate earlier studies in mice which demonstrated that IFNAR signaling in macrophages and microglial cells limited CNS damage in EAE [15,113]. The most likely explanation for our finding is that the upregulation of ISGs is also beneficial in ENMO, and that we enhance this beneficial effect by the injection of I-IFN. This interpretation would be in line with the observation that several of the upregulated ISGs have tissue protective properties, e.g. Psmb9, P2ry6, Gadd45b, and SerpinG1, while others have anti-inflammatory properties, like P2ry6, IL-10, Sectm1b, Bst2, and Timp1 (Table 2). Moreover, both I-IFNs produced within the inflamed CNS and the I-IFNs peripherally injected into the ENMO rats could jointly reduce the neutrophil infiltration triggered by inflammatory cytokines and attenuate the disruption of the blood-brain barrier [124]. This would be especially important in a disease like NMO or ENMO, where neutrophils play an essential role in lesion formation [125,126].

At first glance, there seems to be a discrepancy between the seemingly protective I-IFN signature in ENMO rats culminating in the formation of smaller NMO-like lesions in I-IFN treated ENMO animals, and the formation of larger NMO-like lesions in wildtype mice compared to their IFNAR deficient counterparts, when both were intracerebrally injected with NMO-IgG and complement [16]. However, antibody-dependent cellular cytotoxicity executed by Fc gamma-receptor 3 (Fcgr3)-positive activated microglia, macrophages and neutrophils is an important factor contributing to the formation of astrocyte-destructive lesions in the presence of NMO-IgG and complement [27,28], and neutrophils were found in much lower numbers in the NMO-IgG/complement-injected CNS of the IFNAR deficient mice [16].

The beneficial outcome of I-IFN treatment of ENMO rats also differs from observations in spinal cord slice cultures, in which the addition of IFN-β had no effects on NMO-IgG/complement mediated tissue damage [17]. Most likely, these discrepancies can be explained by differences in treatment duration (3 days in slice cultures, 2 days and less in ENMO) [127], and by the absence of immune effector cells crossing the blood-brain barrier in the slice cultures.

To what extent do our data, which were obtained from spinal cords of rats with T_H_1 cell-induced ENMO reflect the situation of spinal cords of NMO patients, in which T_H_17 cells are thought to play an important role [128], especially since T_H_17 cells have much higher levels of IFNAR1 [119]? First, both in our ENMO model and in human NMO, activated CD4^+^ T cells are found in the CNS parenchyma [28]. Once these cells are within the tissue, it seems to be irrelevant whether they belong to the T_H_1 or T_H_17 subset of cells, since T_H_17 cells undergo phenotypic conversion to interferon-gamma (IFN-γ) producing T_H_1 cells within the CNS [129,130]. Hence, both types of T_H_ cells can provide the cooperative signaling by IFN-γ needed for the effects of I-IFN [131]. Secondly, both in the ENMO model (see above) and in human NMO [30,31], a clear IL-6 signature was found. And last, our microarray analysis of ENMO spinal cords identified Ifi30/GILT as a differentially expressed and upregulated gene, and this molecule also counts among the top 20 upregulated genes in NMO lesions [66].

Hence, it is tempting to speculate that the gene signature seen within the spinal cords of ENMO rats reflects the gene signature of the spinal cords of NMO patients. Why, then, do NMO patients not profit from treatment with I-IFN?

In contrast to our ENMO rats, which received I-IFN as a short-term treatment when their blood-brain barrier was open, NMO patients were treated for a long time once they were in remission [5–12]. Hence, in these patients, I-IFN could also affect the differentiation and expansion of autoimmune T cells [122] and of plasmablasts/B cells. For studies into these aspects of the action of I-IFN, our model is not suitable, since it is based on passive disease induction, i.e. the transfer of high numbers of fully differentiated activated T cells and of NMO-IgG as humoral effector molecules. One particularly important survival factor for B cells is B cell activating factor of the TNF family (BAFF) [132–134], also known as tumor necrosis factor (ligand) superfamily member 13b (TNFSF13b), which is produced by I-IFN-treated astrocytes, neutrophils, and peripheral blood mononuclear cells. Unfortunately, we could not obtain information about BAFF in ENMO from our microarrays, since genetic information about this molecule is only available for humans and mice, but not for rats (iHOP– http://www.ihop-net.org/, retrieved february 04, 2016). However, in humans, elevated serum levels of BAFF are associated with increased B-cell proliferation and improved survival of B lineage cells [135] and could serve as an explanation for the increase in AQP4 antibody titer observed in an NMO patient in the course of IFN-β treatment [7]. Higher BAFF levels are observed in the CSF of AQP4-antibody positive NMO patients [136,137], in the group of I-IFN treated hepatitis C patients progressing to NMO [138,139] or to other types of antibody-associated autoimmune diseases [140–143], and in the serum of patients with other antibody-driven autoimmune diseases like Sjögren´s syndrome [144,145] or systemic lupus erythematosus [146]. Hence, in patients with NMO, the deleterious effects of BAFF on autoaggressive B lineage cells might outweigh the protective effects of I-IFN within the inflamed CNS.

## Supporting Information

S1 Table Immunologically relevant proteins among the 366 upregulated gene products with GenBank accession numbers, grouped according to GO Term pathway analysis. (PDF)

S2 Table Differentially expressed immunologically relevant genes upregulated in spinal cords of Lewis rats with experimental neuromyelitis optica. (PDF)

S3 Table Footprints of the action/production of type I interferons in experimental autoimmune encephalomyelitis. (PDF)

S4 Table Additional references for S1–S3 Tables. (PDF)
