# Supplementary material for: Experimental Neuromyelitis Optica Induces a Type I Interferon Signature in the Spinal Cord
Source: PLoS One. 2016 Mar 18;11(3):e0151244. doi: 10.1371/journal.pone.0151244 (PMC4798752; doi:10.1371/journal.pone.0151244)
Supplement: S1 Table — (PDF) [file pone.0151244.s001.pdf]

S1 Table. Immunologically relevant proteins among the 366 upregulated gene products with GenBank accession numbers, grouped according to GO Term pathway analysis.

GO Term "immune response":

GENBANK_ACCESSION	GENE NAME
NM_138913	2'-5' oligoadenylate synthetase 1A
XM_001053627	Fc fragment of IgE, high affinity I, receptor for; gamma polypeptide
NM_001100836	Fc fragment of IgG, high affinity Ia, receptor (CD64)
M64368	Fc fragment of IgG, low affinity IIb, receptor (CD32); Fc fragment of IgG, low affinity IIa, receptor (CD32)
NM_001008829	RT1 class I, CE14; RT1 class I, CE16; RT1 class Ia, locus A2; RT1 class Ib, locus Cl; RT1 class Ia, locus A1; RT1 class I, A3
NM_001008840	RT1 class I, CE2
NM_001008841	RT1 class I, CE3
AJ004888	RT1 class Ib, locus Aw2
NM_001048045	RT1 class Ib, locus M5
NM_019262	complement component 1, q subcomponent, beta polypeptide
NM_001077642	complement factor D (adipsin)
NM_133624	guanylate binding protein 2
NM_012968	interleukin 1 receptor accessory protein
NM_012854	interleukin 10
NM_022634	leukocyte specific transcript 1
NM_001108076	myosin IF
NM_001106031	nucleoside phosphorylase
NM_012708	proteasome (prosome, macropain) subunit, beta type 9 (large multifunctional peptidase 2)
NM_053908	protein tyrosine phosphatase, non-receptor type 6
NM_017160	ribosomal protein S6; similar to 40S ribosomal protein S6
NM_199093	serine (or cysteine) peptidase inhibitor, clade G, member 1
AF436847	similar to complement factor H-related protein

GENBANK_ACCESSION	GENE NAME
NM_138533	spondin 2, extracellular matrix protein
NM_172328	tachykinin 4
NM_198769	toll-like receptor 2

GO Term "antigen processing and presentation":

GENBANK_ACCESSION	GENE NAME
XM_001053627	Fc fragment of IgE, high affinity I, receptor for; gamma polypeptide
NM_001100836	Fc fragment of IgG, high affinity Ia, receptor (CD64)
M64368	Fc fragment of IgG, low affinity IIb, receptor (CD32); Fc fragment of IgG, low affinity IIa, receptor (CD32)
M64368	Fc gamma receptor II beta
NM_001008829	RT1 class I, CE14; RT1 class I, CE16; RT1 class Ia, locus A2; RT1 class Ib, locus Cl; RT1 class Ia, locus A1; RT1 class I, A3
NM_001008840	RT1 class I, CE2
NM_001008841	RT1 class I, CE3
AJ004888	RT1 class Ib, locus Aw2
NM_001048045	RT1 class Ib, locus M5
NM_001030026	interferon gamma inducible protein 30
NM_012708	proteasome (prosome, macropain) subunit, beta type 9 (large multifunctional peptidase 2)

GO Term "regulation of immune effector processes":

GENBANK_ACCESSION	GENE NAME
XM_001053627	Fc fragment of IgE, high affinity I, receptor for; gamma polypeptide
NM_001100836	Fc fragment of IgG, high affinity Ia, receptor (CD64)
M64368	Fc fragment of IgG, low affinity IIb, receptor (CD32); Fc fragment of IgG, low affinity IIa, receptor

GENBANK_ACCESSION	GENE NAME
	(CD32)
M64368	Fc gamma receptor II beta
NM_001008829	RT1 class I, CE14; RT1 class I, CE16; RT1 class Ia, locus A2; RT1 class Ib, locus Cl; RT1 class Ia, locus A1; RT1 class I, A3
NM_012488	alpha-2-macroglobulin
NM_012854	interleukin 10
NM_001106031	nucleoside phosphorylase
NM_053908	protein tyrosine phosphatase, non-receptor type 6
NM_199093	serine (or cysteine) peptidase inhibitor, clade G, member 1
AF436847	similar to complement factor H-related protein

GO Term "positive regulation of immune responses":

GENBANK_ACCESSION	GENE NAME
XM_001053627	Fc fragment of IgE, high affinity I, receptor for; gamma polypeptide
NM_001100836	Fc fragment of IgG, high affinity Ia, receptor (CD64)
M64368	Fc fragment of IgG, low affinity IIb, receptor (CD32); Fc fragment of IgG, low affinity IIa, receptor (CD32)
M64368	Fc gamma receptor II beta
NM_001008829	RT1 class I, CE14; RT1 class I, CE16; RT1 class Ia, locus A2; RT1 class Ib, locus Cl; RT1 class Ia, locus A1; RT1 class I, A3
NM_019262	complement component 1, q subcomponent, beta polypeptide
NM_001077642	complement factor D (adipsin)
NM_001106031	nucleoside phosphorylase
NM_053908	protein tyrosine phosphatase, non-receptor type 6
NM_199093	serine (or cysteine) peptidase inhibitor, clade G, member 1
AF436847	similar to complement factor H-related protein
NM_198769	toll-like receptor 2

GO Term “defense response”:

**GENBANK_ACCESSION**	**GENE NAME**
XM_001053627	Fc fragment of IgE, high affinity I, receptor for; gamma polypeptide
NM_001100836	Fc fragment of IgG, high affinity Ia, receptor (CD64)
M64368	Fc fragment of IgG, low affinity IIb, receptor (CD32); Fc fragment of IgG, low affinity IIa, receptor (CD32)
NM_031634	Mediterranean fever
NM_012488	alpha-2-macroglobulin
NM_001108452	coactosin-like 1 (Dictyostelium)
NM_019262	complement component 1, q subcomponent, beta polypeptide
NM_001077642	complement factor D (adipsin)
NM_013185	hemopoietic cell kinase
NM_012968	interleukin 1 receptor accessory protein
NM_012854	interleukin 10
NM_001025420	lymphocyte-specific protein 1
NM_001108076	myosin IF
NM_053908	protein tyrosine phosphatase, non-receptor type 6
NM_031531	serine (or cysteine) peptidase inhibitor, clade A, member 3N
NM_199093	serine (or cysteine) peptidase inhibitor, clade G, member 1
AF436847	similar to complement factor H-related protein
NM_198769	toll-like receptor 2
