# Supplementary material for: Experimental Neuromyelitis Optica Induces a Type I Interferon Signature in the Spinal Cord
Source: PLoS One. 2016 Mar 18;11(3):e0151244. doi: 10.1371/journal.pone.0151244 (PMC4798752; doi:10.1371/journal.pone.0151244)
Supplement: S2 Table — (PDF) [file pone.0151244.s002.pdf]

S2 Table. Differentially expressed immunologically relevant genes upregulated in spinal cords of Lewis rats with experimental neuromyelitis optica

Target Id	fc ENMO/ EAE	fc all T / all non-T	Gene symbol	major function	Ref.
NM_031085	432.4	72.4	Prkch	protein kinase C eta; down-regulated through immune responses	[1]
NM_001108076	14.9	11.4	Myo1f_predicted	unconventional myosin type 1F; fusion partner in infant acute monocytic leukemia;	[2]
NM_021989	13.9	1.2	Timp2	tissue inhibitor of metalloproteinase 2; constitutively expressed in astrocytes and serum exposed microglia	[3,4]
NM_001077642	11.8	4.3	Cfd	complement factor D (adipsin); alternative complement pathway; found in astroglioma	[5]
NM_012488	7.8	0.9	A2m	alpha-2-macroglobulin; broad spectrum protease-binding protein; leads to protease clearance; acute phase protein, induced by IL-6.	[6] [7]
NM_019262	7.6	3.7	C1qb	complement factor C1qb; classical pathway; found in microglia and macrophages	[8,9]
NM_001100836	7.5	14.2	Fcgr1	Fc receptor, IgG, high affinity I;	[10]

NM_001106586	6.8	3.1	Irf5_predicted	interferon regulatory factor 5 (predicted); highly expressed in M1 macrophages; promotes polarization of inflammatory macrophages and TH1-TH17 responses.	[11]
NM_001108452	6.4	1.4	Cotl1_predicted	coactosin-like 1; stabilizes 5-lipoxygenase and thus promotes synthesis of pro-inflammatory leukotrienes; found on certain types of Fcgr3 expressing neutrophils;	[12,13]
NM_031657	6.4	1.2	Grk6	G protein-coupled receptor kinase 6; intracellular kinase; regulates sensitivity of G-protein coupled receptors including those involved in migration of inflammatory cells.	[14]
NM_001107578	5.9	0.7	Ms4a1_predicted	membrane-spanning 4-domains, subfamily A, member 1, predicted; = CD20; expressed on B cells	
NM_001030026	5.6	5.1	Ifi30	interferon gamma inducible protein 30; = Gamma-interferon-inducible lysosomal thiol reductase (GILT); found among top 20 upregulated genes in NMO lesions	[15]
**ENSRNOT00000045433**	**5.0**	**0.7**	**ENSRNOT00000045433**	**„Similar to interferon-α“**	
NM_012968	4.0	1.3	Il1rap	interleukin 1 receptor accessory protein;	[10]
NM_212525	3.9	4.7	Tyrobp	TYRO protein tyrosine kinase binding protein; = DAP12;<br>associates with TREM2. TREM2/DAP12 complexes trigger immune responses in macrophages and microglia; phagocytosis	[16,17]

NM_031114	3.8	2.2	S100a10	regulation of plasminogen-dependent macrophage invasion	[18]
XM_001073024	3.2	1.0	LOC690045	similar to immunoreceptor Ly49si	
NM_001105971	3.0	48.2	Slamf9_predicted	SLAM family member 9 (predicted); leukocyte cell surface receptor	[19]
NM_013169	3.0	2.2	Cd3d	component of CD3 complex on T cells	
NM_133624	3.0	83.7	Gbp2	guanylate nucleotide binding protein 2; inhibits cell spreading	[20]
BC061859	2.9	2.9	Tcirg1	T-cell, immune regulator 1; in T cells and macrophages; needed to acidify phagosomes; = TIRC7, recruits Ptpn6	[21,22]
BC061858	2.9	0.8	Ptbp1	polypyrimidine tract binding protein 1; critical for turnover and subcellular distribution of CD40L mRNA	[23]
XM_344628	2.8	0.7	LOC364775	similar to interleukin 15 receptor alpha chain isoform 1C	
NM_198769	2.8	7.5	Tlr2	Toll-like receptor 2;	[10]
NM_138913	2.6	3.6	Oas1a	2'-5' oligoadenylate synthetase 1A; in antiviral signaling cascade	[24]
NM_022634	2.5	4.4	Lst1	leucocyte specific transcript 1; possibly immuno-modulatory	[25]
NM_057124	2.3	15.9	P2ry6	pyrimidinergic receptor P2Y, G-protein coupled, 6; in T cells and macrophages; inhibits activation of effector T cells.	[26,27]
NM_001031638	2.2	1.0	Cd68	on monocytes, macrophages, microglia, neutrophils;	[10]
NM_012854	2.1	0.8	Il10	interleukin 10	[10]

NM_001004226	1.9	1.2	RGD1303130	kidney predominant protein NCU-G1; lysosomal protein;	[27]
NM_001048045	1.9	1.0	RT1-M5	RT1 class Ib, locus M5;	
NM_175577	1.9	1.2	Cd6	CD6 antigen; preferentially activated in T cells; functions as pattern recognition receptor for microbial components; expression dependent on runx3.	[28,29]
S79307	1.9	0.9	S79307	immunoglobulin heavy chain [rats, humanized lympholytic MoAb CAMPATH-1H, mRNA, 1465 nt].	
NM_001105989	1.9	0.75	Cr2_predicted	complement receptor 2	[10]
XM_573983	1.9	1.2	Tgfbi	transforming growth factor, beta, induced;	[10]
NM_053383	1.8	1.0	C1qr1	complement factor C1qr1; classical pathway; in microglia	[30]
NM_001109509	1.8	1.2	Gpr84	G protein-coupled receptor 84; on microglia; induced by pro-inflammatory cytokines like IL-1 and TNF-α;	[31]
NM_001105840	1.8	1.1	Rab5c_predicted	member RAS oncogene family predicted; in macrophages; induced by IL-6; needed for phagosome maturation	[32]
NM_031609	1.8	1.2	Nbl1	neuroblastoma, suppression of tumorigenicity 1 (Nbl1); = DAN; secreted antagonist of bone morphogenic protein; acts as inhibitor of monocyte chemotaxis	[33]
NM_053953	1.8	1.0	Il1r2	interleukin 1 receptor, type II (Il1r2)	[10]
NM_001106554	1.7	1.0	Il1f6_predicted	interleukin 1 family, member 6 (predicted) (Il1f6_predicted)	[10]

NM_053372	1.7	1.7	Slpi	secretory leukocyte peptidase inhibitor; associated with MMP-2 and MMP-9 to promote migration; inhibits neutrophil apoptosis	[34,35]
NM_001105811	1.7	1.3	Rilp_predicted	Rab interacting lysosomal protein	[36]
NM_053908	1.7	2.5	Ptpn6	protein tyrosine phosphatase, non-receptor type 6; = Shp-1; in neutrophils, dendritic cells, macrophages; controls neutrophil numbers and survival; regulates IL-6 and IL-1 production	[37-39]
XM_344357	1.6	1.2	XM_344357	similar to T cell receptor V alpha 8.5;	
AJ004888	1.6	1.7	RT1-Aw2	MHC class Ib;	
NM_031634	1.6	4.2	Mefv	Mediterranean fever; expressed in mature neutrophils and is predominantly upregulated in response to IFN-γ or TNF-α, but can also be induced by IFN-α	[40]
XM_001053627	1.6	2.1	LOC498279	similar to high affinity immunoglobulin epsilon receptor gamma-subunit precursor (FcεRIg); when complexed with immunoglobulin-like transcript 7 and when signaling is initiated via this complex, production of IFN and proinflammatory cytokines by plasmacytoid dendritic cells is inhibited	[41]
XM_227483	1.6	1.1	Etv3	ets variant gene 3 (ETV3), upregulated by interleukin 10; similar to BXMAS1-like protein 2;	[42]
NM_001007622	1.4	1.6	Pdlim2	PDZ and LIM domain 2 (Pdlim2), in macrophages; overexpression associated with enhanced adhesion and increased nuclear activity of NF-kappaB; limits the production of proinflammatory cytokines	[43] [44]

M87786	1.4	1.6	M87786	immunoglobulin light chain variable region, complementarity-determining region, partial	
NM_030833	1.4	2.2	Ifitm2	interferon induced transmembrane protein 2; anti-viral	[45]
AF436847	1.4	1.0	Cfh	Complement factor H; has complement inhibitory activity	[46]
NM_001008840	1.3	1.7	RT1-CE2	RT1 class I, CE2;	
NM_130425	1.3	1.3	Runx3	runt-related transcription factor 3; one of its functions: regulation of CD6 expression	[29]
ENSRNOT 00000059672	1.3	1.1	ENSRNOT 00000059672	„similar to granzyme N“	
NM_176077	1.2	1.0	G6pc3	glucose 6 phosphatase, catalytic, 3; essential for neutrophil function and survival	[47]
NM_001008384	1.2	1.5	Rac2	RAS-related C3 botulinum substrate 2; required for the formation of neutrophil extracellular traps	[48]
NM_053492	1.2	3.8	Cdw92	CDW92 antigen, transcript variant 2; stably expressed on monocytes; regulates specific subsets of dendritic cells	[49]
NM_001007008	0.4	0.4	Ndg2	Nur77 downstream gene 2 (Ndg2); implicated in negative selection of thymocytes; Nur77-mediated T cell apoptosis involves Bcl-2 independent transcriptional activation of apoptotic pathways	[50]
NM_001109175	0.4	0.7	Ccno	cyclin O; responsible for apoptosis induction in lymphoid cells	[51]

XM_344339	0.3	1.8	XM_344339	T cell receptor V-alpha J-alpha (LOC364314)	

fold changes (fc) above 1 indicate an upregulation in gene expression, fc below 1 indicate a downregulation.

EAE = mean value of EAE_{coI} and EAE_{coP} ; all T = mean value of all T cell mediated diseases (i.e. ENMO, EAE_{coI}, EAE_{coP})

all non-T = mean value of all non-inflammatory controls (i.e. healthy control animals, animals injected with NMO-IgG only, animals injected with control IgG only.
