# Supplementary material for: Experimental Neuromyelitis Optica Induces a Type I Interferon Signature in the Spinal Cord
Source: PLoS One. 2016 Mar 18;11(3):e0151244. doi: 10.1371/journal.pone.0151244 (PMC4798752; doi:10.1371/journal.pone.0151244)
Supplement: S3 Table — (PDF) [file pone.0151244.s003.pdf]

S3 Table. Footprints of the action/production of type I interferons in experimental autoimmune encephalomyelitis.

Target Id	fc EAE_{coP} (*: EAE_{coI} instead of EAE_{coP}) / all non-T	Gene symbol
NM_133624	72.9	GBP2
CF111193	28.7	B2m
NM_012708	9.4	Psmb9
NM_199093	3.7	Serpin G1
NM_001030026	3.4	Ifi30
NM_001011921	3.2	PDGFRL
NM_013069	3.0	CD74
NM_138913	2.5	Oas1a
NM_030833	2.3	Ifitm2
NM_139341	1.9	Slc15a3
BQ196649	*1.7	Gpx2
NM_198134	1.6	Bst2
NM_001024755	1.6	Ube2l6
NM_001008321	1.5	Gadd45b
NM_001109514	1.4	Slc25a28
NM_001013895	*1.4	Prkd2
NM_057124	1.3	P2ry6
NM_001037353	1.1	Timp1

Type I interferon stimulated genes in EAE were identified using the list of 31 type I interferon stimulated genes identified in ENMO (see table 3).

Fold changes (fc) > 1 indicate an upregulation in gene expression.

all non-T = mean value of all non-inflammatory controls (i.e. healthy control animals, animals injected with NMO-IgG only, animals injected with control IgG only).
