# Supplementary material for: Experimental Neuromyelitis Optica Induces a Type I Interferon Signature in the Spinal Cord
Source: PLoS One. 2016 Mar 18;11(3):e0151244. doi: 10.1371/journal.pone.0151244 (PMC4798752; doi:10.1371/journal.pone.0151244)
Supplement: S4 Table — (PDF) [file pone.0151244.s004.pdf]

S4 Table: Supplementary references

1. Takata Y, Hamada D, Miyatake K, Nakano S, Shinomiya F, Scfe CR, et al. (2007) Genetic association between the PRKCH gene encoding protein kinase Ceta isozyme and rheumatoid arthritis in the Japanese population. Arthritis Rheum 56: 30-42.
2. Taki T, Akiyama M, Saito S, Ono R, Taniwaki M, Kato Y, et al. (2005) The MYO1F, unconventional myosin type 1F, gene is fused to MLL in infant acute monocytic leukemia with a complex translocation involving chromosomes 7, 11, 19 and 22. Oncogene 24: 5191-5197.
3. Agapova OA, Ricard CS, Salvador-Silva M, Hernandez MR (2001) Expression of matrix metalloproteinases and tissue inhibitors of metalloproteinases in human optic nerve head astrocytes. Glia 33: 205-216.
4. Welser-Alves JV, Crocker SJ, Milner R (2011) A dual role for microglia in promoting tissue inhibitor of metalloproteinase (TIMP) expression in glial cells in response to neuroinflammatory stimuli. J Neuroinflammation 8: 61.
5. Barnum SR, Ishii Y, Agrawal A, Volanakis JE (1992) Production and interferon-gamma-mediated regulation of complement component C2 and factors B and D by the astroglioma cell line U105-MG. Biochem J 287 (Pt 2): 595-601.
6. Armstrong PB, Quigley JP (1999) Alpha2-macroglobulin: an evolutionarily conserved arm of the innate immune system. Dev Comp Immunol 23: 375-390.
7. Taniguchi T, Takaoka A (2001) A weak signal for strong responses: interferon-alpha/beta revisited. Nat Rev Mol Cell Biol 2: 378-386.
8. Pasinetti GM, Hassler M, Stone D, Finch CE (1999) Glial gene expression during aging in rat striatum and in long-term responses to 6-OHDA lesions. Synapse 31: 278-284.
9. Van Beek J, Chan P, Bernaudin M, Petit E, MacKenzie ET, Fontaine M (2000) Glial responses, clusterin, and complement in permanent focal cerebral ischemia in the mouse. Glia 31: 39-50.
10. Hoffmann R, Valencia A (2004) A gene network for navigating the literature. Nat Genet 36: 664.
11. Krausgruber T, Blazek K, Smallie T, Alzabin S, Lockstone H, Sahgal N, et al. (2011) IRF5 promotes inflammatory macrophage polarization and TH1-TH17 responses. Nat Immunol 12: 231-238.
12. Yokoyama T, Kobayashi T, Yamamoto K, Yamagata A, Oofusa K, Yoshie H (2010) Proteomic profiling of human neutrophils in relation to immunoglobulin G Fc receptor IIIb polymorphism. J Periodontal Res 45: 780-787.
13. Esser J, Rakonjac M, Hofmann B, Fischer L, Provost P, Schneider G, et al. (2010) Coactosin-like protein functions as a stabilizing chaperone for 5-lipoxygenase: role of tryptophan 102. Biochem J 425: 265-274.
14. Eijkelkamp N, Heijnen CJ, Lucas A, Premont RT, Elsenbruch S, Schedlowski M, et al. (2007) G protein-coupled receptor kinase 6 controls chronicity and severity of dextran sodium sulphate-induced colitis in mice. Gut 56: 847-854.
15. Satoh J, Obayashi S, Misawa T, Tabunoki H, Yamamura T, Arima K, et al. (2008) Neuromyelitis optica/Devic's disease: gene expression profiling of brain lesions. Neuropathology 28: 561-576.
16. Paloneva J, Mandelin J, Kiialainen A, Bohling T, Prudlo J, Hakola P, et al. (2003) DAP12/TREM2 deficiency results in impaired osteoclast differentiation and osteoporotic features. J Exp Med 198: 669-675.
17. Paloneva J, Manninen T, Christman G, Hovanes K, Mandelin J, Adolfsson R, et al. (2002) Mutations in two genes encoding different subunits of a receptor signaling complex result in an identical disease phenotype. Am J Hum Genet 71: 656-662.
18. O'Connell PA, Surette AP, Liwski RS, Svenningsson P, Waisman DM (2010) S100A10 regulates plasminogen-dependent macrophage invasion. Blood 116: 1136-1146.
19. Fennelly JA, Tiwari B, Davis SJ, Evans EJ (2001) CD2F-10: a new member of the CD2 subset of the immunoglobulin superfamily. Immunogenetics 53: 599-602.

20. Messmer-Blust AF, Balasubramanian S, Gorbacheva VY, Jeyaratnam JA, Vestal DJ (2010) The interferon-gamma-induced murine guanylate-binding protein-2 inhibits rac activation during cell spreading on fibronectin and after platelet-derived growth factor treatment: role for phosphatidylinositol 3-kinase. Mol Biol Cell 21: 2514-2528.
21. Sun-Wada GH, Tabata H, Kawamura N, Aoyama M, Wada Y (2009) Direct recruitment of H+-ATPase from lysosomes for phagosomal acidification. J Cell Sci 122: 2504-2513.
22. Bulwin GC, Walter S, Schlawinsky M, Heinemann T, Schulze A, Hohne W, et al. (2008) HLA-DR alpha 2 mediates negative signalling via binding to Tirc7 leading to anti-inflammatory and apoptotic effects in lymphocytes in vitro and in vivo. PLoS One 3: e1576.
23. Matus-Nicodemos R, Vavassori S, Castro-Faix M, Valentin-Acevedo A, Singh K, Marcelli V, et al. (2011) Polypyrimidine tract-binding protein is critical for the turnover and subcellular distribution of CD40 ligand mRNA in CD4+ T cells. J Immunol 186: 2164-2171.
24. Austin BA, James C, Silverman RH, Carr DJ (2005) Critical role for the oligoadenylate synthetase/RNase L pathway in response to IFN-beta during acute ocular herpes simplex virus type 1 infection. J Immunol 175: 1100-1106.
25. Mulcahy H, O'Rourke KP, Adams C, Molloy MG, O'Gara F (2006) LST1 and NCR3 expression in autoimmune inflammation and in response to IFN-gamma, LPS and microbial infection. Immunogenetics 57: 893-903.
26. Lattin JE, Schroder K, Su AI, Walker JR, Zhang J, Wiltshire T, et al. (2008) Expression analysis of G Protein-Coupled Receptors in mouse macrophages. Immunome Res 4: 5.
27. Giannattasio G, Ohta S, Boyce JR, Xing W, Balestrieri B, Boyce JA (2011) The purinergic G protein-coupled receptor 6 inhibits effector T cell activation in allergic pulmonary inflammation. J Immunol 187: 1486-1495.
28. Sarrias MR, Farnos M, Mota R, Sanchez-Barbero F, Ibanez A, Gimferrer I, et al. (2007) CD6 binds to pathogen-associated molecular patterns and protects from LPS-induced septic shock. Proc Natl Acad Sci U S A 104: 11724-11729.
29. Arman M, Aguilera-Montilla N, Mas V, Puig-Kroger A, Pignatelli M, Guigo R, et al. (2009) The human CD6 gene is transcriptionally regulated by RUNX and Ets transcription factors in T cells. Mol Immunol 46: 2226-2235.
30. Lynch NJ, Willis CL, Nolan CC, Roscher S, Fowler MJ, Weihe E, et al. (2004) Microglial activation and increased synthesis of complement component C1q precedes blood-brain barrier dysfunction in rats. Mol Immunol 40: 709-716.
31. Bouchard C, Page J, Bedard A, Tremblay P, Vallieres L (2007) G protein-coupled receptor 84, a microglia-associated protein expressed in neuroinflammatory conditions. Glia 55: 790-800.
32. Bhattacharya M, Ojha N, Solanki S, Mukhopadhyay CK, Madan R, Patel N, et al. (2006) IL-6 and IL-12 specifically regulate the expression of Rab5 and Rab7 via distinct signaling pathways. EMBO J 25: 2878-2888.
33. Chen B, Blair DG, Plisov S, Vasiliev G, Perantoni AO, Chen Q, et al. (2004) Cutting edge: bone morphogenetic protein antagonists Drm/Gremlin and Dan interact with Slits and act as negative regulators of monocyte chemotaxis. J Immunol 173: 5914-5917.
34. Subramaniyam D, Hollander C, Westin U, Erjefalt J, Stevens T, Janciauskiene S (2011) Secretory leukocyte protease inhibitor inhibits neutrophil apoptosis. Respirology 16: 300-307.
35. Choi BD, Jeong SJ, Wang G, Park JJ, Lim DS, Kim BH, et al. (2011) Secretory leukocyte protease inhibitor is associated with MMP-2 and MMP-9 to promote migration and invasion in SNU638 gastric cancer cells. Int J Mol Med 28: 527-534.
36. Colucci AM, Spinosa MR, Bucci C (2005) Expression, assay, and functional properties of RILP. Methods Enzymol 403: 664-675.
37. Nesterovitch AB, Szanto S, Gonda A, Bardos T, Kis-Toth K, Adarichev VA, et al. (2011) Spontaneous insertion of a b2 element in the ptpn6 gene drives a systemic autoinflammatory disease in mice resembling neutrophilic dermatosis in humans. Am J Pathol 178: 1701-1714.
38. Rego D, Kumar A, Nilchi L, Wright K, Huang S, Kozlowski M (2011) IL-6 production is positively regulated by two distinct Src homology domain 2-containing tyrosine phosphatase-1 (SHP-1)-dependent CCAAT/enhancer-binding protein beta and NF-kappaB pathways and an SHP-1-

independent NF-kappaB pathway in lipopolysaccharide-stimulated bone marrow-derived macrophages. J Immunol 186: 5443-5456.
39. Ramachandran IR, Song W, Lapteva N, Seethammagari M, Slawin KM, Spencer DM, et al. (2011) The phosphatase SRC homology region 2 domain-containing phosphatase-1 is an intrinsic central regulator of dendritic cell function. J Immunol 186: 3934-3945.
40. Centola M, Wood G, Frucht DM, Galon J, Aringer M, Farrell C, et al. (2000) The gene for familial Mediterranean fever, MEFV, is expressed in early leukocyte development and is regulated in response to inflammatory mediators. Blood 95: 3223-3231.
41. Cao W, Bover L, Cho M, Wen X, Hanabuchi S, Bao M, et al. (2009) Regulation of TLR7/9 responses in plasmacytoid dendritic cells by BST2 and ILT7 receptor interaction. J Exp Med 206: 1603-1614.
42. El Kasmi KC, Smith AM, Williams L, Neale G, Panopoulos AD, Watowich SS, et al. (2007) Cutting edge: A transcriptional repressor and corepressor induced by the STAT3-regulated anti-inflammatory signaling pathway. J Immunol 179: 7215-7219.
43. Healy NC, O'Connor R (2009) Sequestration of PDLIM2 in the cytoplasm of monocytic/macrophage cells is associated with adhesion and increased nuclear activity of NF-kappaB. J Leukoc Biol 85: 481-490.
44. Tanaka T, Grusby MJ, Kaisho T (2007) PDLIM2-mediated termination of transcription factor NF-kappaB activation by intranuclear sequestration and degradation of the p65 subunit. Nat Immunol 8: 584-591.
45. Schoggins JW, Wilson SJ, Panis M, Murphy MY, Jones CT, Bieniasz P, et al. (2011) A diverse range of gene products are effectors of the type I interferon antiviral response. Nature 472: 481-485.
46. Ito S, Tamura N (1983) Inhibition of classical C5 convertase in the complement system by factor H. Immunology 50: 631-635.
47. Cheung YY, Kim SY, Yiu WH, Pan CJ, Jun HS, Ruef RA, et al. (2007) Impaired neutrophil activity and increased susceptibility to bacterial infection in mice lacking glucose-6-phosphatase-beta. J Clin Invest 117: 784-793.
48. Lim MB, Kuiper JW, Katchky A, Goldberg H, Glogauer M (2011) Rac2 is required for the formation of neutrophil extracellular traps. J Leukoc Biol 90: 771-776.
49. Wille S, Szekeres A, Majdic O, Prager E, Staffler G, Stockl J, et al. (2001) Characterization of CDw92 as a member of the choline transporter-like protein family regulated specifically on dendritic cells. J Immunol 167: 5795-5804.
50. Rajpal A, Cho YA, Yelent B, Koza-Taylor PH, Li D, Chen E, et al. (2003) Transcriptional activation of known and novel apoptotic pathways by Nur77 orphan steroid receptor. EMBO J 22: 6526-6536.
51. Roig MB, Roset R, Ortet L, Balsiger NA, Anfosso A, Cabellos L, et al. (2009) Identification of a novel cyclin required for the intrinsic apoptosis pathway in lymphoid cells. Cell Death Differ 16: 230-243.
